# Sustainable paper manufacture from *Cladophora via* hydrochloric acid process

**DOI:** 10.1039/d6su00225k

**Published:** 2026-09-07

**Authors:** Karl Mihhels, A. Topias Kilpinen, Neptun Yousefi, Roozbeh Abidnejad, Jouni Paltakari, Erlantz Lizundia, Jaanika Blomster, Eero Kontturi

**Affiliations:** a Department of Bioproducts and Biosystems, Aalto University, School of Chemical Engineering 02150 Espoo Finland eero.kontturi@aalto.fi; b Department of Chemical Engineering, Imperial College London South Kensington Campus, Exhibition Rd London SW7 2AZ UK n.yousefi@imperial.ac.uk; c Life Cycle Thinking Group, Department of Graphic Design and Engineering Projects, Faculty of Engineering in Bilbao, University of the Basque Country (EHU) Bilbao 48013 Spain; d Department of Environmental Sciences, University of Helsinki Finland

## Abstract

As global demand for wood resources is projected to exceed five billion m^3^ by 2050, developing sustainable non-forest alternatives for paper production is an urgent industrial priority. We demonstrate a single-stage conversion of raw *Cladophora* biomass into papermaking fibres using aqueous hydrochloric acid at ambient conditions. The acid treatment yields cellulose-rich fibres that can be formed into sheets by standard papermaking methods, and it generates a spent liquor from which hydrochloric acid can be recovered by distillation. The recovered algal fibres form dense networks with mechanical properties comparable to commercial high-yield wood pulps. The environmental impacts of processing algal paper sheets at lab-scale were quantified using Life Cycle Assessment. Our study establishes a direct and operationally simple route for producing functional fibre networks from *Cladophora*, and it clarifies the sustainability limits and opportunities associated with papermaking from algae.

## Abstract 2

HCl pulping converts algae into paper with properties comparable to wood-based paper. Environmental impact of manufacturing is assessed and compared to relevant alternatives.

## Graphical abstract

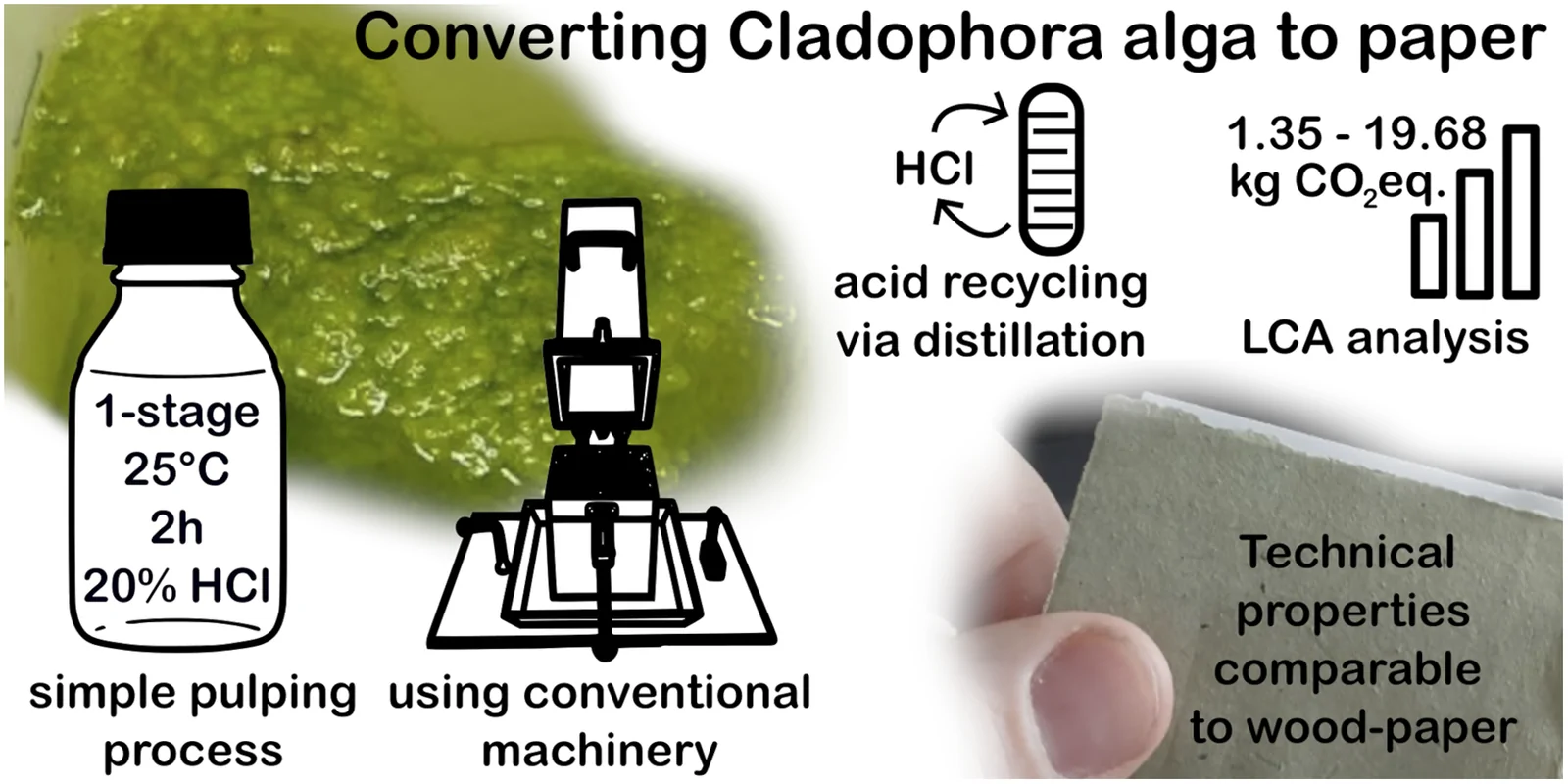

**Sustainability spotlight**

This research introduces acid pulping to convert algal biomass into paper with performance comparable to terrestrial biomass paper. The environmental cost of production of this algal paper is assessed and benchmarked against relevant materials, focusing on chemical recycling's impact and feasibility. The work presents a potential replacement for a commodity good, advancing SDG 9 (Industry, Innovation and Infrastructure) and SDG 12 (Responsible Consumption and Production), while creating opportunities in SDG 13 (Climate Action), SDG 14 (Life Below Water) and SDG 15 (Life on Land) by converting marine biomass, currently an environmental pollutant, into a valuable product, reducing wood use for papermaking.

## 1 Introduction

Paper remains a cornerstone material for global industry and daily use, yet nearly all production still relies on wood as the primary feedstock, with 99.5% of capacity derived from forest resources.^1^ Seaweeds represent an underexplored reservoir of biomass, and green seaweeds of the order Cladophorales are particularly compelling due to their exceptionally high cellulose content^2–4^ and their overabundance in eutrophicated waters worldwide.^5–9^ As wood use is estimated to increase from 4 billion m^3^ to 4.25–5.25 billion m^3^ by 2050 and, as our planet is still undergoing deforestation on a global level,^10^ developing alternatives to substitute forest resources is essential for sustainable development worldwide.

*Cladophora glomerata* was selected for this study due to its local abundance in the Baltic Sea, its cellulose-rich composition characteristic of the Cladophorales,^2–4^ and prior compositional characterisation in our earlier work.^11^ Other cellulose-rich algae, such as red algae of the genus *Gelidiella*,^12^ exist but are generally less suited to conventional papermaking due to differing cell structure. Furthermore, *C. glomerata* and other filamentous green algae of the order Cladophorales, accumulate and form substantial standing stocks in eutrophicated waters. Estimates report an annual standing stock of approximately 170 kt in the Laurentian Great Lakes region^13^ and growth densities of up to 300 g m^−2^ in the Neva estuary of the eastern Baltic Sea.^14^ This biomass currently accumulates as a relatively unmanaged nuisance product of eutrophication, with no established harvesting infrastructure or commercial utilisation. The scale and recurring seasonal availability of this feedstock therefore represent a substantial, currently untapped resource for valorisation into papermaking fibre. Removal of this bloom biomass is expected to be broadly beneficial for eutrophication management, since it also removes the nitrogen and phosphorus bound within the algal tissue, positioning papermaking valorisation as complementary to sustainable management of these waters.

Unfortunately, there is a limited amount of information available on attempts at algal papermaking. Sporadic accounts of pulping and papermaking for some individual algal species^15–18^ often mixed with conventional wood pulp during papermaking^19–21^ can be found, but even in topical reviews^12^ conventional papermaking is not considered a potential use case for algal cellulose. Our research addresses this blind spot directly, focusing attention on this undervalued material application.

Most attempts to convert algal biomass into paper pulp have used some variation of alkaline pulping, albeit exceptions exist.^15–21^ A consistent problem with these methods is a lack of quantitative sustainability assessment. Even when mechanical performance of algal paper is comparable to wood-based papers,^15–17,21,22^ the absence of environmental metrics prevents meaningful benchmarking. This challenge is magnified by the compositional plasticity of algal biomass, which introduces further uncertainty into environmental burden estimates. Consequently, future studies of algal materials must include harmonised environmental indicators to define their authentic sustainability.^23^ This work uses Life Cycle Assessment (LCA) to provide a structured framework for quantifying environmental impacts across material and process stages. Standardized under ISO 14040/14044 and increasingly applied to emerging bio-based systems,^24–26^ LCA can quantify multiple impact categories including climate change potential, eutrophication, acidification, water use, resource criticality, and circularity.^27^

In this work, raw *C. glomerata* biomass is converted into paper using one-stage, room-temperature pulping with HCl below its azeotropic concentration with water. The reasoning for this is threefold: First, algal biomass of *C. glomerata* does not need to be delignified, as it contains no lignin, so there is no reason to prefer alkaline pulping methods that have been developed with lignin removal in mind. Second, it is practical for lab-scale paper pulp production with minimal specialised processing equipment; for example, no pressurized cooking vessels with temperature control are needed. Finally, below the azeotropic concentration, hydrochloric acid can be recycled by evaporative distillation, a method well established on an industrial scale in steel pickling operations.^28–31^ This combination of simplicity, with respect to both pulping and chemical recovery, is something rather unique in the field of algal papermaking and, in our opinion, deserves the attention of the scientific community. Comparatively, developing the chemical recycling of alkaline pulping for algal biomass might prove more challenging due to the high ash content of this biomass. It should be noted that this treatment does not constitute pulping in the conventional, delignification-based sense, since *C. glomerata* contains no lignin to remove. Instead, the acid is likely to have several functionalities: hydrolysing structural carbohydrates,^32^ degrading and partially dissolving proteins,^33^ and solubilising acid-soluble ash components.^34^ The glycosidic bonds are broken predominantly within the amorphous, less-ordered polysaccharide fractions that bind the algal tissue together, while the crystalline cellulose microfibrils are largely preserved. This selectivity is consistent with reports that cellulose from *C. glomerata* is unusually resistant to acid hydrolysis^11^ relative to terrestrial cellulose, and is the likely reason a coherent, papermaking-grade fibre is recovered rather than a fully degraded product.

## 2 Materials and methods

### 2.1 Materials

*C. glomerata* was harvested on 27 August 2024 from coastal waters near the Tvärminne research facility by the Baltic Sea (59°50′40″ N, 23°14′57″ E). Species identification was performed on-site with the limnological expertise of co-author Dr Jaanika Blomster, using the criteria reported in the SI and later confirmed by microscopy. The biomass was mechanically pressed using a hydraulic press (Vares, 18 L, 2 t) to remove excess water and then air-dried at elevated temperature (∼60 °C). A qualitative purification step was carried out after air-drying: pure *C. glomerata* was observed to dry into a fluffy, airy mass, whereas material containing other algae or plant detritus entangled layers of *C. glomerata* biomass around it, forming clumps that could readily be separated and removed. This step removed a minimal fraction (∼1%) of the total harvested material. The dry matter content (DMC) of the air-dry algal biomass was determined following the NREL standard method (60 °C, 18 h).^35^ HCl (aq) (37 weight percent (wt%), Sigma-Aldrich, ACS reagent-grade) was diluted with deionized water (Milli-Q-grade) to prepare 19.6 wt% working solution. NaOH (aq) (1 M, VWR, Titripur®-grade) was used for acid–base titration to determine the exact acid concentration. All reagents were used as received.

#### 2.1.1 Preparation of algal pulp

Air-dried *C. glomerata* biomass was immersed in 19.6 wt% HCl at a solid-to-liquid ratio of 1 : 10 for 2 h at 25 °C in 1 L glass vessels. The vessels were agitated at 120 rpm on an orbital lab shaker. After treatment, excess acid was removed by filtration through a ceramic Büchner funnel, with the algal biomass forming the filtration mat. The recovered acid filtrate was collected, and its density and acid concentration were determined as described in the SI. The algal pulp was then diluted with tap water and washed using a polyethylene mesh bag (200 µm) at a flow rate of 1.4 L min^−1^ for 12 h. The resulting algal pulp was pressed and mixed by hand. Moisture was let to equalize in a sealed plastic bag for 24 h before analysis.

#### 2.1.2 Preparation of algal paper sheets

Algal pulp was diluted to 1.5% consistency and disintegrated according to ISO 5263-2 standard,^36^ using two intensities: 300 and 10 000 revolutions. These settings correspond to low and standard mechanical forces used to break up fibre flocs and homogenize the pulp in conventional papermaking. Paper sheets were formed from disintegrated pulps following ISO 5269-1 standard.^37^ After pressing and before conditioning, the paper sheets, still attached to the supporting blotter papers, were dried using a Lorentzen & Wettre FI 119 drying drum at 60 °C for 4 h. The paper samples are hereafter referred to as “Alga 300” (300 revolution disintegration) and “Alga 10k” (10 000 revolution disintegration). Local tap water served as process water for all stages of the papermaking.

#### 2.1.3 Preparation of wood-based paper sheets

Reference sheets were produced from peroxide-bleached, pressure-groundwood pulp from Finnish spruce (sourced from a Finnish paper mill). The pulp was disintegrated according to ISO 5263-2 standard,^36^ and paper sheets were formed following ISO 5269-1 standard.^37^ After pressing and before conditioning, the paper sheets, still attached to the supporting blotter papers, were dried using a Lorentzen & Wettre FI 119 drying drum at 60 °C for 4 h. These samples are hereafter referred to as “MechPulp”. Local tap water served as process water for all stages of the papermaking.

### 2.2 Methods

#### 2.2.1 Chemical characterisation of algal pulp and paper

Dry matter content, ash content, and the carbohydrate content of algal papers were determined according to relevant NREL standards.^35,38^ Prior to analysis, all samples were equilibrated in standard-conditioned space (50% RH, 25 °C) for a minimum of 24 h to ensure measurement consistency.

#### 2.2.2 Testing of paper sheets

##### 2.2.2.1 Morphology

The structure of the sheets was examined utilising a field-emission scanning electron microscope (Zeiss Sigma VP, Germany) operated at 5 kV accelerating voltage. Before imaging, samples were sputter-coated with a 6 nm Au/Pd layer using a Leica EM ACE600 high-vacuum coater.

##### 2.2.2.2 Paper sheet characterisations

Thickness, density, and specific volume (bulk) of the paper sheets were measured according to ISO 534.^39^ Sample mass was determined utilising a four-digit analytical balance, and thickness was measured with a Lorentzen & Wettre SE 250 D thickness tester. Grammage was calculated according to ISO 536.^40^

Mechanical properties were evaluated according to ISO 1924-2 standard^41^ using a Lorentzen & Wettre SE 064 tensile tester to determine tensile strength, tensile index, strain at break, tensile energy absorption (TEA), and TEA index.

Bendtsen air permeability of the sheets was measured with a Lorentzen & Wettre SE 114 air permeability and roughness tester according to ISO 5636-3 standard.^42^ ISO brightness and opacity of algal paper sheets were determined according to ISO 2470-1 standard^43^ using the Elrepho Lorentzen & Wettre SE 070 spectrophotometer. Results for ISO brightness are considered indicative only, as the green colour of the sheets falls outside the intended range of the standard for white and near-white samples.

All paper quality tests were performed on five replicates of “Alga 300” and “Alga 10k” and ten replicates of “MechPulp”. Prior to testing, sheets were conditioned for two weeks under standard atmospheric conditions (23 °C, 50% relative humidity) as specified in ISO 187 standard.^44^

### 2.3 Life cycle assessment

The LCA methodology was utilised to determine the environmental impacts of raw cellulose isolation from *C. glomerata* and its processing into paper sheets. The model employed a cradle-to-grave system boundary that includes raw material sourcing, on-site electricity consumption, waste management, and end-of-life (EoL) treatment. Equipment amortization was not included. The life cycle inventory (LCI), derived from laboratory-scale experiments, is provided in the SI (Fig. S7 and Table S4).

System expansion with substitution has been implemented for the co-products in accordance with ISO 14044. To align the assessments with the European Union renewable-energy targets (EU/2023/2413), a renewable medium-voltage electricity mix was applied as the source of electricity. Background data was drawn primarily from the databases “ecoinvent v3.11 Cut-Off Unit Processes” (released in December 2024) and “Agribalyse 3.1.1” (released in January 2024). Alternative sources are explicitly identified in the LCI or where cited. Calculations were performed in OpenLCA 2.5 software using the Environmental Footprint v3.1 impact assessment method.

Two functional units were applied for impact normalization. First, a mass-based functional unit was used, which allows results to be obtained for 1 kg of material. This provides a common basis for comparison with other materials. To facilitate comparisons between materials serving the same final function, we considered the mechanical performance delivered by the algal paper to obtain the impacts per specific tensile strength as:^45,46^1
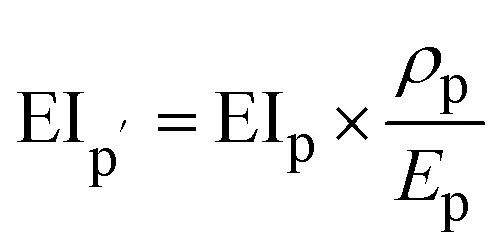
where *ρ*_p_ represents the density (kg dm^−3^), *E*_p_ the tensile modulus (GPa), and EI_p_ is the environmental impact per kg. The usage of functional units for comparing mechanical properties of materials in the context of LCA, and the justifications of use of Young's modulus for this purpose have been discussed, for example, by Furberg *et al.*^47^

For benchmarking, cradle-to-grave impacts were also calculated for three types of wood based paper: tissue paper, super calendered (SC) paper, and uncoated wood-free (UWF) paper (Note: UWF refers to wood based paper where lignin has been chemically removed), and five plastic materials, including raw material sourcing, processing (sheet formation or extrusion), incineration and fossil CO_2_ emissions released during combustion.

To estimate the water consumption for the LCA analysis, we chose a wash ratio of 3.6 times the reaction volume, an estimate slightly above the industrial displacement-washing benchmark of 2.5,^48^ rather than the substantially higher water consumption observed under non-optimized laboratory conditions.

## 3 Results and discussion

### 3.1 Characterisation of algal papers

#### 3.1.1 Pulping of algae

The pulping process was developed through preliminary trials described in the SI (Fig. S1–S5 and Table S1 and accompanying text). Mechanistically, this step is best understood as acidic degradation of structural carbohydrates, proteins and acid-soluble ash rather than delignification-type pulping. With respect to carbohydrates, HCl preferentially cleaves glycosidic linkages in disordered polysaccharide regions, including the heteropolysaccharide fraction and amorphous domains of cellulose itself, while crystalline cellulose, which is unusually resistant to acid hydrolysis in *C. glomerata* relative to terrestrial celluloses, is largely retained. This selective hydrolysis explains why a cellulose-rich, fibrous, papermaking-usable pulp is obtained under these conditions rather than extensive biomass dissolution.

**Fig. 1:**
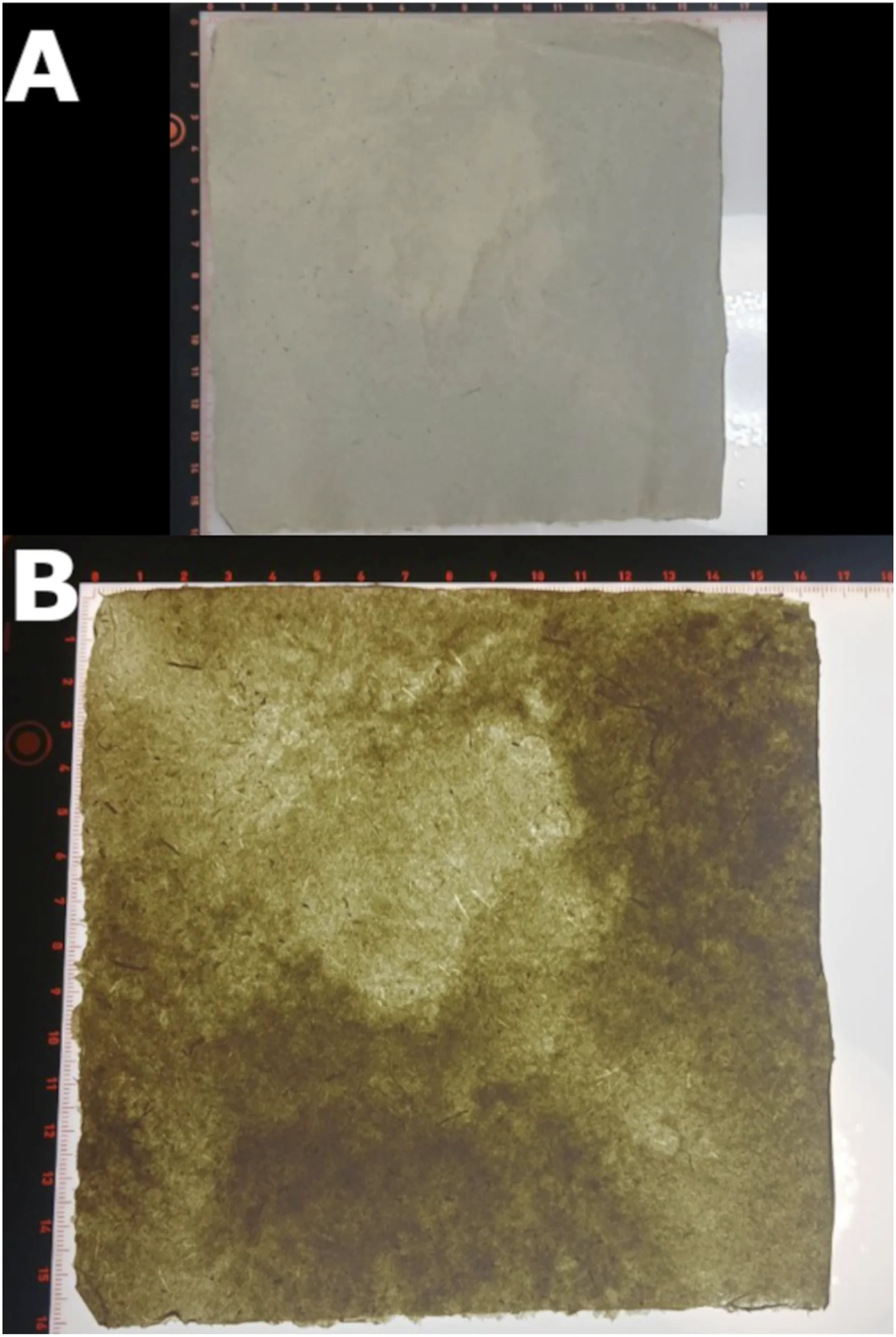
Representative *C. glomerata*-based paper sheet produced by the methodology used in this publication on a light pad (5000 lux, 6000K) for visual representation of paper and paper formation. In panel (A) light pad is turned off, in panel (B) on. The visual appearance of formation in (B) is affected by the residual colour and low opacity of the unbleached pulp, which can exaggerate apparent non-uniformity; the absence of visible floc particles indicates adequate fibre dispersion during sheet formation, sufficient to produce coherent, testable sheets.

The pulping method used in the main study (2 h immersion of air-dry algal biomass in 19.4 wt% HCl at room temperature) produced fibres that formed coherent sheets using standard papermaking methods and enabled recovery of HCl from the spent acid by simple distillation. Fig. 1 shows a representative algal paper sheet on a light pad. The green colour in the paper is most likely caused by traces of chlorophyll from the original paper and can be removed by conventional bleaching if necessary. (Fig. S3 and S4 and accompanying text in SI).

Before the pulping of algae, we measured the concentration of HCl solution by titration and found it to be 19.6 ± 0.1 wt% (95% confidence). The air-dried algae contained 9.8 ± 0.1 wt% (95% confidence) moisture, which lowers the effective acid concentration in the reaction medium to 19.4 wt% HCl. After the reaction, titration of the spent liquor showed an HCl concentration of 18.5 ± 0.1 wt% (95% confidence). This decrease corresponds to a consumption of 10.6 g of pure HCl per 100 g of air-dried algae. Most of this acid consumption is likely caused by neutralisation of acid by the CaCO_3_ present in the algal biomass, as discussed in previous work.^11^

**Fig. 2:**
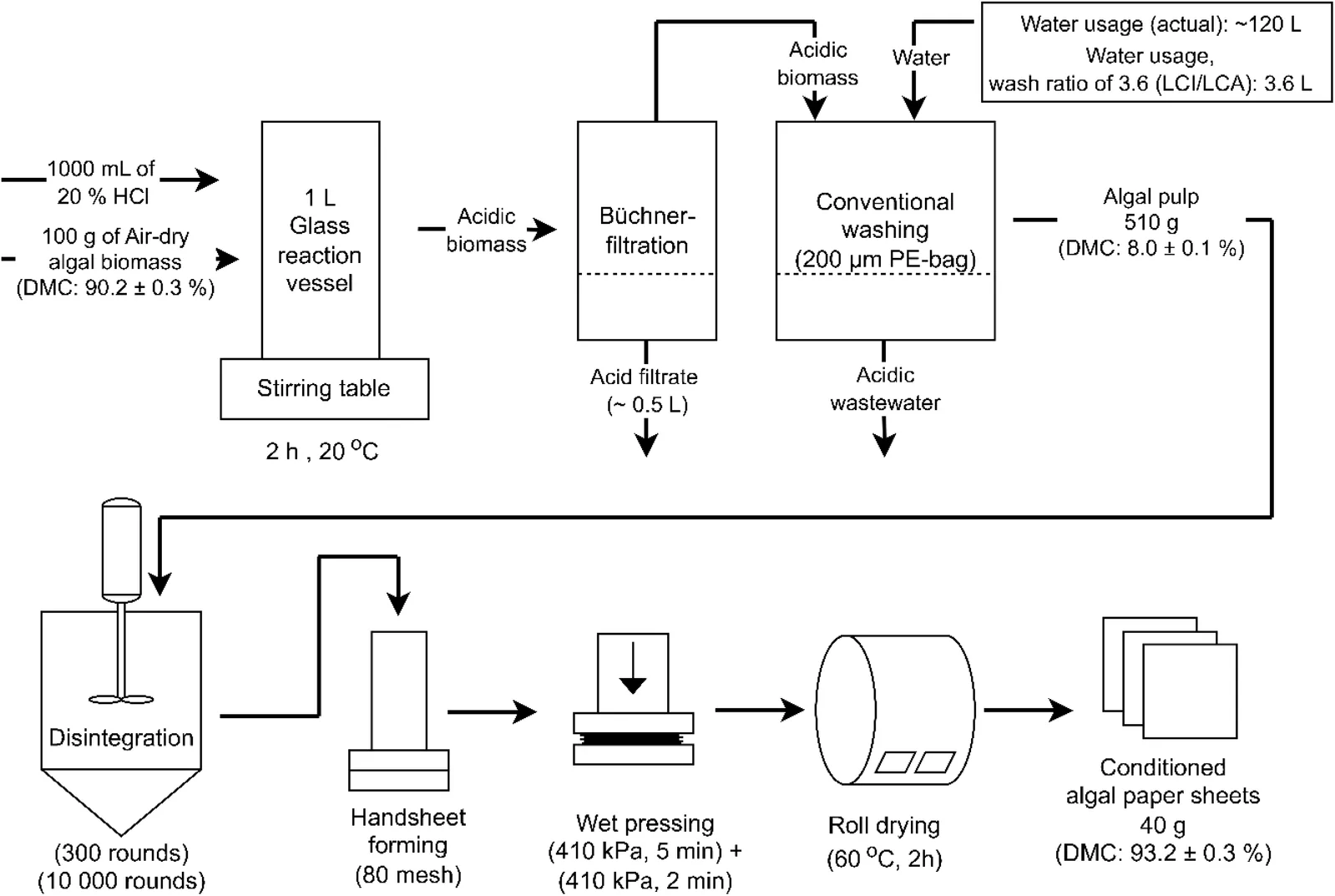
Process flow and mass balance for the conversion of *C. glomerata* into paper sheets. The schematic illustrates the single-stage hydrochloric acid pulping process in its entirety. Measured masses and dry-matter contents (DMC) are provided at each stage to define the laboratory-scale material balance. The mass balance has been normalized for 100 g, while total amount of air-dry algae used in experimental work to form this mass balance was 840 g.

The yield of washed algal pulp was 41% (oven-dry basis) relative to the raw biomass. Yield losses during disintegration and sheet formation were negligible, with the dry weight of the formed sheets matching that of the pulp used to produce them within 1–3%. Fig. 2 shows the process flow chart for the lab-scale pulping and papermaking.

Büchner filtration produced an acid filtrate containing the dissolved components, but the upper layers of the biomass cake remained visibly wet even after extended filtration (15 min). The washing efficiency of the laboratory setup was extremely low compared to what can be achieved by dedicated washing equipment. As a result, a large volume of water was required (∼1000 L), for the total amount of air-dry algal biomass pulped in this work, generating substantial quantities of mildly acidic wastewater. In industrial papermaking, displacement washers typically operate with a wash ratio of ∼2.5 (washing-liquid volume to original liquid volume) to obtain clean pulp.^48^ This assumption is our best estimation of realistic washing water consumption based on our experience with practical laboratory and LCA work, but other wash ratios (in the range of 3–10) could also have suited this purpose. We would also like to draw attention to the fact that after washing, the fibres remain well dispersed and do not get stuck to papermaking machinery surfaces. This property is characteristic of the acid pulping presented in this work, and it is not an inherent property of *C. glomerata* pulps (Fig. S5 and accompanying text).

#### 3.1.2 Chemical composition of the algal papers

The chemical composition of *C. glomerata* papers is summarized in Table 1. Both “Alga 300” and “Alga 10k” samples contained roughly 67% cellulose and 5% residual heteropolysaccharides. The two samples had very similar chemical compositions, with a small (∼1%) difference in ash content between Alga 10k and Alga 300 that may tentatively reflect mechanical detachment of small inorganic particles during longer disintegration, though this difference is close to measurement uncertainty and should not be overinterpreted. For detailed chemical composition of the raw *C. glomerata* biomass harvested from the same location, see earlier work by Mihhels *et al.*^11^

**Table 1:** Chemical composition of *C. glomerata* papers

Paper sample	Moisture content^a^ (%)	Cellulose fraction^b^ (%)	Ash fraction (%)	Heteropolysaccharides^c^ fraction (%)	Undetermined components fraction^d^ (%)
Alga 300	6.5 ± 0.5	68.1 ± 1.1	12.7 ± 0.2	5.1 ± 0.1	∼7.6
Alga 10k	7.1 ± 0.1	66.3 ± 1.1	11.3 ± 0.3	5.4 ± 0.1	∼9.9

a Moisture content reported from samples kept under standard condition (50% RH, 25 °C).

b The cellulose content is estimated to be the wt% of glucose recovered from the sample.

c The weight% of the heteropolysaccharides fraction is defined as the sum of arabinose, rhamnose, galactose, xylose, and mannose content identified in the sample. Individual sugar yields are provided in the SI (Table S2).

d Incomplete mass closure is common in analysis of algal biomass^49,50^ and is noted in state-of-the-art analytical guidelines.^51^ Uncertainties represent 95% confidence interval.

Pigments, chiefly chlorophyll, were not quantified separately, as they constitute a minor mass fraction and are not expected to meaningfully affect mechanical performance. They are responsible for the green colouration of the unbleached algal papers (Fig. 1), a consequence of the pulp not being bleached in this study rather than an inherent material property, as shown by the bleaching trials in the SI (Fig. S3 and S4). A faint odour was noted in the air-dried biomass, which intensified somewhat on prolonged storage; this odour was eliminated during HCl pulping and washing, with no detectable smell remaining in the resulting algal pulp or paper.

#### 3.1.3 Paper properties of the algal papers and the reference wood sample

**Fig. 3:**
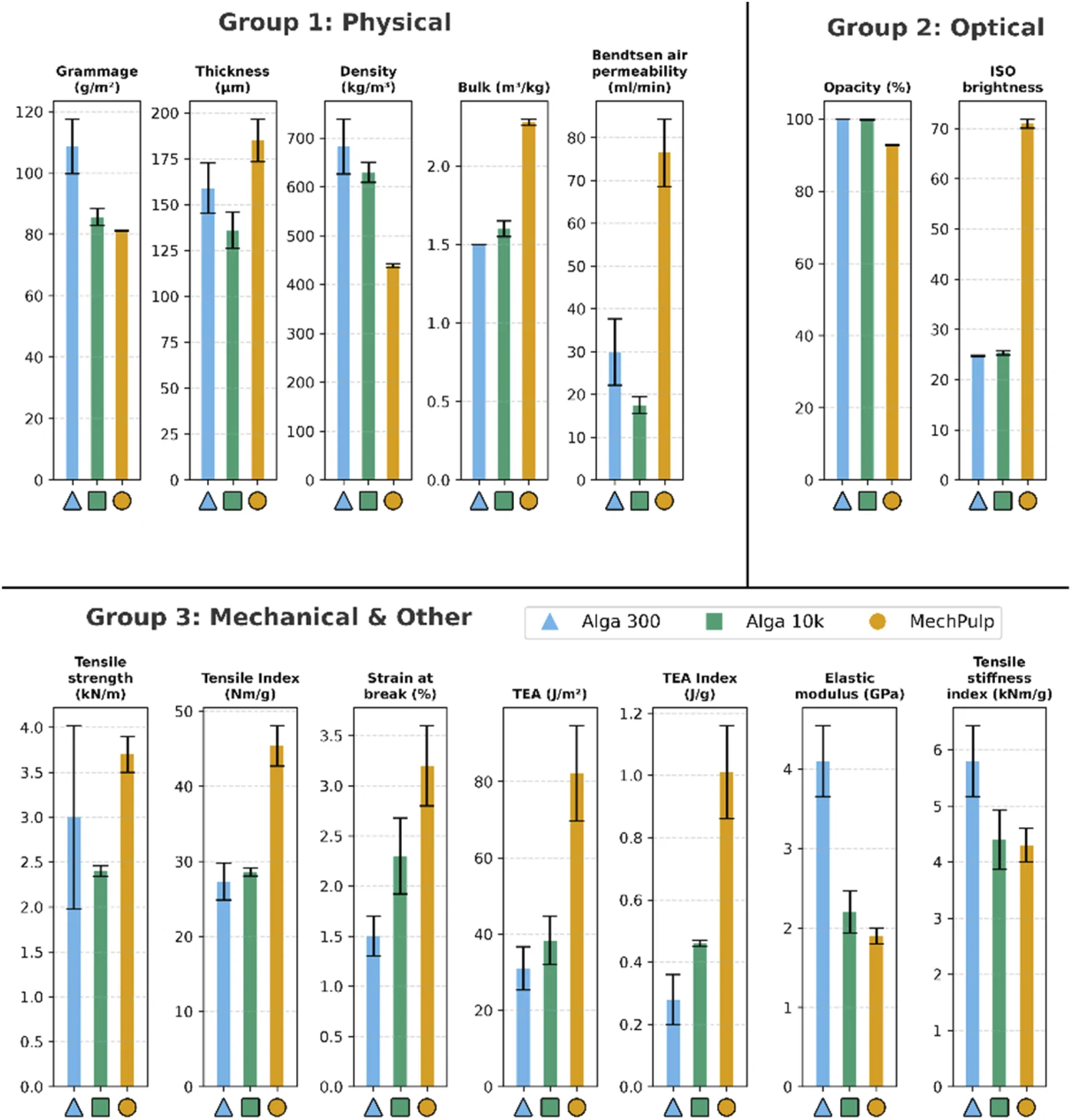
Comparative physical, optical, and mechanical properties of algal and wood-based papers. Data is presented for algal papers disintegrated for 300 rotations (blue triangle) and 10 000 rotations (green square), compared against spruce-derived mechanical groundwood paper (orange circle). Error bars denote 95% confidence intervals based on five replicates for algal samples and ten for wood-based references. Numeric datasets are provided in the SI (Table S3).

Measured paper properties have been compiled in Fig. 3. The “Alga 300” samples exhibited higher variance in nearly all measurements than the “Alga 10k” group, suggesting that extended disintegration is vital for achieving uniform paper properties. Grammage differed somewhat between sample sets (108.6, 85.6 and 81.1 g m^−2^ for Alga 300, Alga 10k and MechPulp respectively), most likely reflecting a combination of measurement variability and moisture-content inhomogeneity in the pulp used for handsheet formation. As mechanical properties are reported here as grammage-normalised (indexed) values, this variance does not affect the validity of the comparisons drawn.

While the tensile index of the algal papers was approximately 60% of the reference wood-fibre papers (27.3–28.6 Nm g^−1^*vs.* 45.4 Nm g^−1^), their mechanical strength remains comparable to sheets from commercial unbeaten pulps (34 Nm g^−1^).^52^ Consequently, algal paper is a viable candidate for low-end paper and cardboard grades, particularly if further refining or additives are used.

**Fig. 4:**
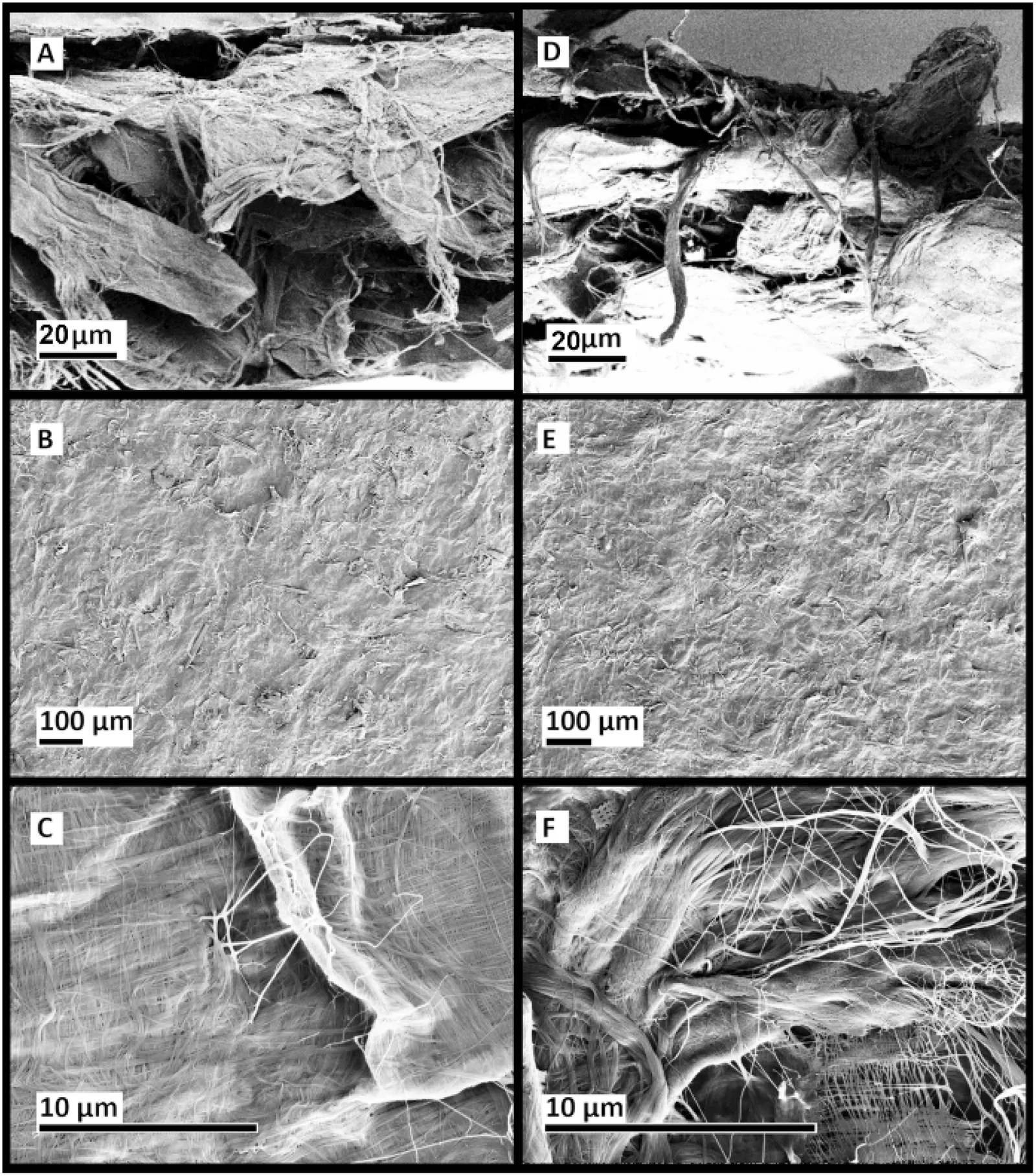
SEM micrographs of algal paper sheets at varying disintegration intensities. Panels (A–C) illustrate algal paper subjected to 300 disintegration cycles (Alga 300), while panels (D–F) show paper after 10 000 cycles (Alga 10k). Frames (A) and (D) present cross-sectional views, whereas (B, C, E and F) display paper surfaces. High-magnification images (C and F) reveal dense fibre networks with distinct nanofibres and nanofibre bundles derived from the algal cell wall.

Notably, the mildly disintegrated “Alga 300” sheets showed higher stiffness (5.8 kNm g^−1^) compared to both “Alga 10k” and wood-based samples (4.3–4.4 kNm g^−1^). This increased stiffness may be attributed to a lower degree of fibrillation, as the more intact fibre matrix observed in the SEM images (Fig. 4) provides greater resistance to deformation.

Both algal papers exhibited lower strain at break but higher tensile energy absorption index than the wood-based reference, reflecting lower extensibility but greater stiffness and resistance to deformation. The algal sheets were also denser, less air-permeable and opaquer than the wood-based reference samples. ISO brightness of the algal papers was low, consistent with the natural green colouration of the sheets (Fig. 1) and the absence of bleaching. Preliminary experiments (Fig. S3, S4 and Table S1 and accompanying text) show that the algal pulp responds to conventional hydrogen peroxide bleaching. The Schopper–Riegler number measuring pulp drainage (°SR) was not determined for the algal pulps, as its applicability to algal pulps has not been established. Qualitative observations made during the drainage of algal papers from preliminary trials have been included in the Section 1.2 of SI.

#### 3.1.4 SEM microscopy of the algal paper

Individual fibres are difficult to discern from the images, except for some fibres sticking out of the fracture surfaces in the cross-section images (Panels A & D in Fig. 4). Compared to typical wood paper structures, the algal paper is very smooth and no voids between the fibres are visible. This is most likely a combination of the natural morphology of algal cells and the relatively high content of ash (11.3–12.7%) in the paper, with the native ash in the algal biomass behaving as a filler during papermaking. The presence of this ash-based filler and the subsequent lack of porosity also explains the lower air permeability of the algal papers. Both silica-based ash and fibre fines likely contribute to this effect: *C. glomerata* cell walls are known to contain silica,^53^ a major contributor to the paper's ash content that can act as a filler, while some fraction of true fibre fines, differing in nature from wood pulp fines given the distinct algal cell structure, is also likely present. Distinguishing the relative contribution of each to the observed low air permeability would require dedicated further study.

If we focus purely on the fibre surfaces, SEM analysis revealed clear morphological distinctions between “Alga 300” and “Alga 10k” sheets. The Alga 300 sample is characterised by largely intact fibre surfaces, whereas the Alga 10k sample exhibits some broken or disintegrated fibre surfaces with extensive fibrillation. These structural variations are most evident when comparing panels (B) and (E), which demonstrate increased fibrillation-induced surface roughness, and panels (C) and (F), which highlight more intense cell wall deformations and increased separation of nanofibres from the primary wall structure.

The higher tensile stiffness observed in the Alga 300 samples likely stems from these intact fibres forming a stiffer matrix compared to the highly fibrillated 10k samples. While this remains a qualitative observation, it provides a plausible structural basis for the mechanical findings. Further research is required to definitively quantify the relationship between algal cell wall fibrillation and macro-scale paper properties.

### 3.2 Environmental impact determination

#### 3.2.1 LCA of algal paper

The environmental impacts originating from the valorisation of algae into cellulosic films were estimated through Life Cycle Assessment.^24,54^ Pulp and paper mills are among the largest biorefineries in operation worldwide, converting a single biomass feedstock into a primary product alongside multiple co-products and process residues; the algal pulping process described here fits within this same framing, particularly for integrated pulp-and-paper mill operation.

Applying LCA to biorefinery systems is inherently complex because product, co-products and wastes must all be accounted for, and each modelling choice requires meticulous explanation and transparent justification.^55,56^ It is therefore necessary to clarify the key assumptions underlying both the background and foreground systems.

In modelling raw algae, a zero-burden approach was not adopted. According to ISO 14044, wastes are “substances or objects which the holder intends or is required to dispose of”. Although current algae blooms are mainly considered to be environmental nuisances requiring mitigation,^57^ the algae are harvested here as a feedstock for a value-added product. Consequently, upstream impacts such as cultivation and harvesting must be included to provide meaningful insight into the use of this biomass as an alternative papermaking raw material.^58^

Reported environmental impacts of seaweed cultivation vary widely, with values of 0.1550 CO_2_-eq. kg^−1^ wet-weight for *Saccharina latissima* grown in Ireland (no biogenic carbon accounting),^59^ 0.0522 CO_2_-eq. kg^−1^ fresh weight for cultivation in Sweden (biogenic carbon accounting),^60^ and 0.0820 CO_2_-eq. kg^−1^ wet weight in Norway (biogenic carbon accounting).^61^ These impacts are far lower than those associated with algae production for direct food consumption, which range from 5.60 to 5.76 CO_2_-eq. kg^−1^ dehydrated algae (*Himanthalia elongata*, *Ulva intestinalis*, and *Fucus serratus*, Agribalyse 3.1.1 database and EF 3.1 assessment). For this study, we consider cultivation impacts of *C. glomerata* for papermaking to be closer to an industrial raw material cultivation (*e.g*., alginate from *Saccharina latissima*) than food-grade material cultivation. Accordingly, assuming 10 wt% dry matter in wet algae, the acquisition impacts were modeled as 2.75% of those reported for *Ulva lactuca* intended for food consumption (a species forming joint blooms with *C. glomerata*)^62^ with data taken from Agribalyse 3.1.1, without biogenic carbon accounting.

**Fig. 5:**
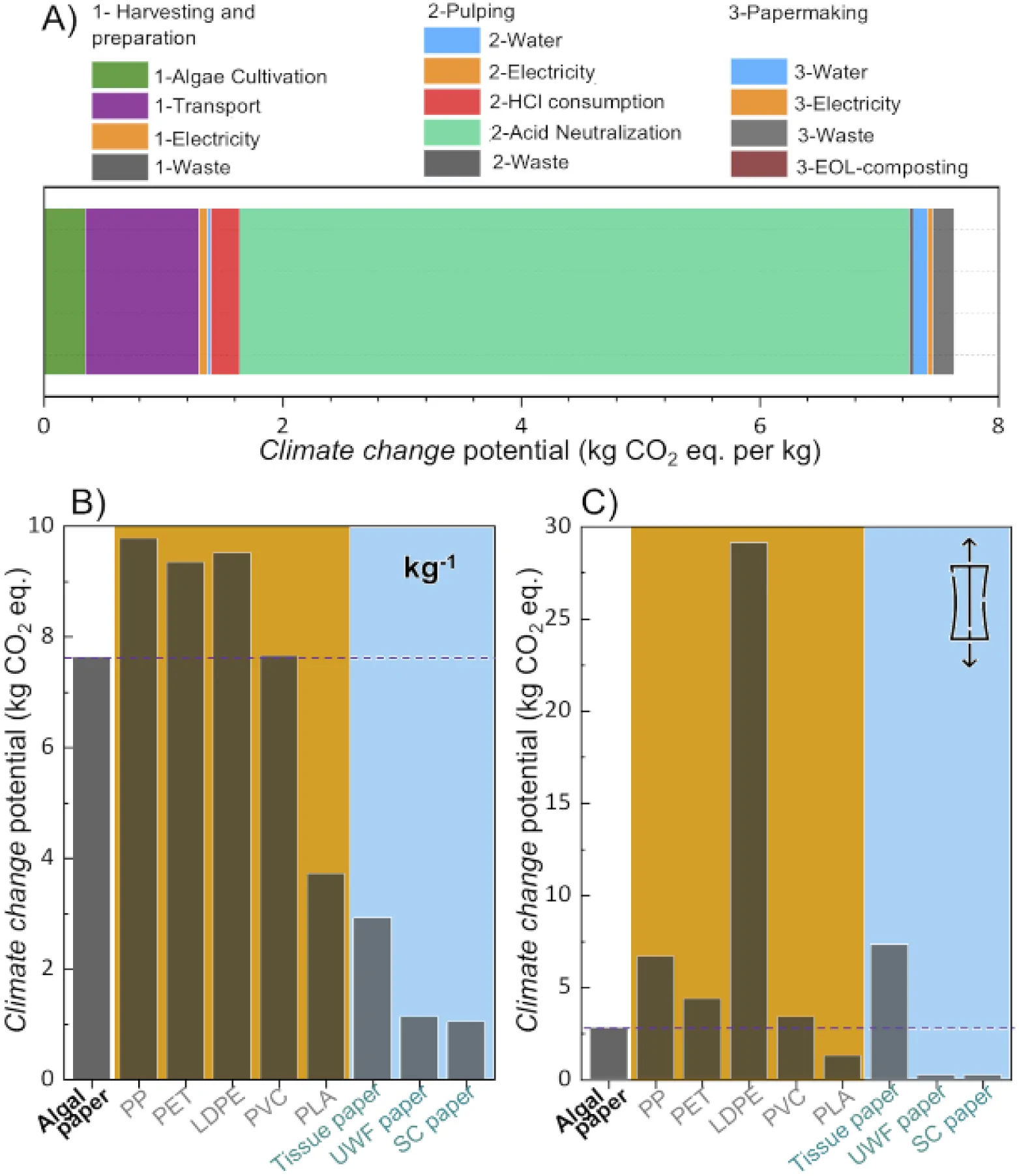
Life cycle climate change potential of *C. glomerata* paper. (A) Disaggregated cradle-to-grave carbon footprint for algal paper processing, expressed in kilograms of CO_2_ equivalents per kilogram of material. (B) Comparative analysis with benchmark materials per unit mass. (C) Performance-based comparison against benchmark materials, incorporating specific density and tensile modulus to reflect technical end-use. In panels (B) and (C), fossil-derived plastics are highlighted in orange, while lignocellulosic materials are shown in light blue. Data sources (Tables S5, S8 and Fig. S8) and complementary analysis using other environmentally relevant indicators with comparisons to wood-paper (Fig. S9) reported in SI.

The climate change potential (per kilogram of material) has been assessed firstly because of its policy relevance and widespread use, which facilitates comparison with other petroleum-derived and bio-based materials. As shown by the disaggregated cradle-to-grave climate change potential in Fig. 5A, algal paper fabrication from *C. glomerata* bears a footprint of 7.64 CO^2^-eq. kg^−1^. The soda ash needed to neutralize the 18.5 wt% HCl acid filtrate during the second processing step is the main impact driver, while algae cultivation and its transport to the processing plant are the next environmental hotspots. To provide context, Fig. 5B compares the obtained cradle-to-grave climate change potential with benchmark fossil-derived plastic and lignocellulosic-biomass-derived paper materials. The results reveal that the algal paper bears a lower footprint than polypropylene (PP), polyethylene terephthalate (PET), and low-density polyethylene (LDPE), and a very similar one to polyvinyl chloride (PVC). Unfortunately, the algal-paper shows a considerably larger climate change potential over traditional, wood-based lignocellulosic-biomass-derived paper products (cradle-to-grave), which have footprints from 0.52 to 2.92 CO_2_-eq. kg^−1^. However, a large part of this higher carbon footprint originates from the inefficiencies inherent in small-scale operations (laboratory-scale processing), where our process lacks the heat and water recovery systems employed by industrial processes (thus resulting in higher energy and chemical use per kilogram of output material).

The climate change potential (per kilogram of material) has been assessed because of its policy relevance and widespread use, which facilitates comparison with other petroleum-derived and bio-based materials. For this analysis, the energy consumption of disintegration has been calculated according to the alga 10k procedure.

Given their compositional similarities, the differences in impact between algae-based and lignocellulosic biomass paper were calculated for eight additional categories, and the results are shown in the SI (Fig. S10). The algal paper performs worse in all the categories studied, a result in line with the high-resource intensity of lab-scale manufacturing. This reflects the absence of the heat and water recovery systems, process integration, and incremental efficiency gains present in the mature industrial processes used to produce the wood-based benchmark materials, rather than an inherent limitation of algal biomass as a papermaking feedstock; comparable or improved performance is expected upon process optimisation and upscaling. Considering that algae cultivation of *C. glomerata* does not require freshwater, fertilizers or arable land (reducing the risk of deforestation and land transformation that is inherently related with conventional lignocellulosic sources), we foresee comparable or even improved impacts across all categories upon process upscaling. If we consider the intended end-use of the material, *e.g*. manufacturing of technical materials like packaging, Fig. 5C, shows the climate change potential based on the second functional unit according to eqn (1), which considers the specific density and Young's modulus of the material (1.5 g cm^−3^, 4.1 GPa, respectively). In this metric, algal paper offers a clear improvement over most conventional plastic materials and can even compete against lignocellulosic-biomass derived tissue paper.

**Fig. 6:**
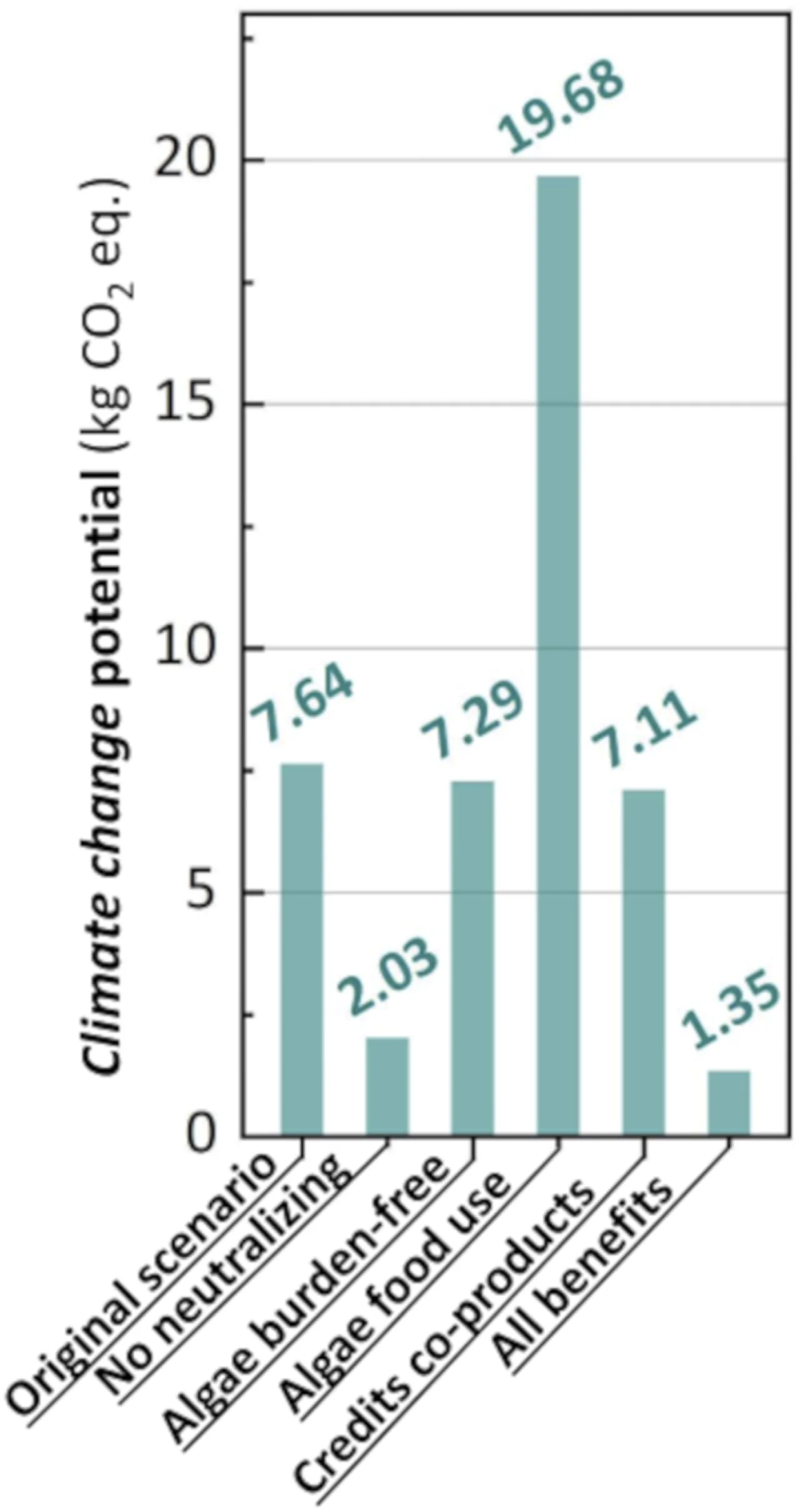
Sensitivity analysis results considering different multifunctionality allocation methods for algal paper processing from *C. glomerata* expressed in kilograms of CO_2_ equivalents per kilo of material (cradle-to-grave).

Finally, the impact of allocation should be noted in the LCA. If we assume that the process fractions currently being disposed of as waste (*e.g.* algal biomass dissolved in acid during pulping) are taken advantage of, this affects significantly the obtained LCA results.^56,62^ Therefore, a sensitivity analysis under alternative scenarios was conducted to evaluate the applied multifunctionality allocation methods. These results are summarized in Fig. 6.

If the algal biomass is considered burden-free (as currently this biomass accumulates in the sea after the growing season to form the green tide)^63^ the climate change potential is reduced to 7.29 CO_2_-eq. kg^−1^. However, if algae fit for human consumption is used for material processing, a value of 19.68 CO_2_-eq. kg^−1^ is obtained. In addition, if one assumes recovery of acid-dissolved components, where proteins, sulfates, CaCO_3_, and SiO_2_ co-products can be valorised (system expansion with substitution can be found in Table S6 in the SI),^56,63^ a climate change potential of 7.11 CO_2_-eq. kg^−1^ is reached for 1 kg of algal paper. If the largest single contributor to the carbon footprint of the algal paper (soda ash used for neutralizing excess acid) is discounted, the impacts are decreased to 2.03 CO_2_-eq. kg^−1^. When all abovementioned benefits are applied together, a final footprint of 1.35 CO_2_-eq. kg^−1^ for 1 kg of algal paper could be achieved.

Comparatively, literature reports a cradle-to-gate global warming potential of 1.25–14.19 CO_2_-eq. kg^−1^ per kilogram of packaging material derived from marine biorefinery utilising a green energy mix.^64^ These figures vary based on allocation methods, with economic allocation yielding the lowest values by displacing impacts to co-products.^65^ Furthermore, a cradle-to-gate assessment of seaweed-based bioplastic indicates carbon footprint of 2.30 and 3.79 CO_2_-eq. kg^−1^ for composting/substitution, and incineration scenarios, respectively.^66^

#### 3.2.2 Assessment on recycling of HCl

As the carbon footprint of algal paper is largely defined by the usage of soda ash for treatment of excess acid (5.61 CO_2_-eq. kg^−1^ out of 7.64 CO_2_-eq. kg^−1^) we consider it appropriate to suggest reasonable industrial alternatives to lab-scale waste treatment. As the algal pulping process here uses HCl below its azeotropic point, recycling excess acid is possible by simple distillation. Distillation of spent pulping liquor relevant for HCl-recycling (“Acid filtrate” from Fig. 2) was conducted successfully in a laboratory-scale experiment (see SI Fig. S6 and accompanying text), with the acid-dissolved solids from the acid filtrate forming a undistillable, tar-like residue on the bottom of the distillation bulb.

Technologically, the lab-scale distillation performed in this study is roughly similar to the evaporative recovery of acid from the pickling solutions^28–31^ used in the steel industry. A visual comparison and a detailed discussion regarding the validity of this benchmark are provided in Fig. S10 and S11. Our primary assumption is that acid/dissolved biomass solids behave similarly to dissolved metals (acting as inert solids) regarding distillation energy consumption in HCl recovery.

In theory, using a distillation column with infinite reflux can separate HCl into its azeotrope concentration and pure distilled water. In practice, energy efficiency must be balanced against the quantity of recovered HCl. To provide a preliminary estimate of the environmental impact of HCl recycling, we used field data from an industrial recycling system supplier (see Fig. S11).^31^

The manufacture of 1 kg of algal paper uses 5.73 kg of neat HCl, of which 0.26 kg is consumed during pulping, leaving 5.47 kg to enter the recycling stream. Assuming the algal pulp could be washed with a wash ratio of 2.5 (comparable to wood pulp)^48^ rather than the factor of 3.6 used in our primary LCA, we can estimate energy intensity by scaling HCl-recycling data from the pickling process.^35^ This indicates total energy consumption of 88 kWh per 1 kg of algal paper. Using the EU medium voltage renewable energy mix as an energy source, this results in an impact of 1.08 CO_2_-eq. kg^−1^ with respect to algal paper produced.

Rounding to the nearest order of magnitude, 1 CO_2_-eq. kg^−1^ represents, in our opinion, minimum possible resource intensity of the HCl-recycling using best currently available technology. This estimate excludes the neutralizing of residual acid or the recovery of dissolved solids, as these steps allow for multiple technical approaches and evaluating these is beyond the scope of this work. Nevertheless, it is highly probable that the impact of HCl-recycling using this methodology remains significantly lower than of NaOH neutralisation. As the distillation of bulk HCl is likely the most energy-intensive stage, these results justify further investigation into this type of acid recovery for algal papermaking.

## 4 Conclusions

Raw *C. glomerata* biomass was converted into papermaking fibres using aqueous hydrochloric acid at ambient conditions with a yield of 41% on dry weight basis. These fibres were processed into paper sheets using two disintegration intensities and benchmarked against conventional mechanical groundwood pulp. The resulting algal papers comprised of 66–68 wt% cellulose, 11–13 wt% ash, 7 wt% water, and 5 wt% of hemicellulose. While increased disintegration reduced property variance, lightly disintegrated algal fibres exhibited improved stiffness compared to both heavily disintegrated algal and wood-based references. Although the tensile index of the algal papers (27.3–28.6 Nm g^−1^) was lower than that of the wood reference paper (45.4 Nm g^−1^), it was comparable to unbeaten market wood pulps. The algal paper sheets also demonstrated significantly higher densities and opacities than wood papers.

Life cycle assessment results for lab-scale production yielded a value of 7.64 CO_2_-eq. kg^−1^ for 1 kg of algal paper produced. This value fluctuates based on feedstock grade and assumed production synergies, ranging from 1.35 to 19.68 CO_2_-eq. kg^−1^. While algal paper is currently outperformed by industrially produced conventional paper, it remains environmentally competitive with most plastics, especially when specific density and tensile modulus are considered. As acid neutralisation is the primary impact driver, industrial HCl-recovery (modelled on steel pickling distillation processes) represents a surmountable path toward enhanced sustainability. Algal paper is therefore a viable candidate as a drop-in replacement for, or additive to, conventional papermaking pulp, particularly for packaging and cardboard grades, given its comparable strength and higher opacity/density relative to wood-based reference paper.

Ultimately, this study establishes an operationally simple route for producing functional fibre networks from *C. glomerata* and clarifies the associated sustainability limits. Our results highlight *C. glomerata* as a credible alternative cellulose source with promising material properties. Transitioning this concept to industrial relevance will require further work, but we see that there are substantial gains to be made. In doing so, this work directly supports SDG 9 and SDG 12, and contributes toward the aims of SDG 13, SDG 14 and SDG 15, by converting an underused marine biomass into a valuable material while reducing pressure on wood-based papermaking resources. This work is in our opinion a clear step towards enabling seaweed-derived cellulose to contribute to sustainable material systems of the future.

## Author contributions

Conceptualization K. M., E. K., N. Y., J. P.; methodology K. M., J. P., J. B., E. K.; investigation K. M., A. T. K., R. A.; resources J. P., J. B., E. K.; data curation K. M.; writing – original draft preparation K. M., E. L.; LCA studies K. M., E. L.; writing-review and editing K. M., A. T. K., N. Y., R. A., J. P., E. L., J. B., E. K.; supervision N. Y. and E. K. All authors have read and agreed to the published version of the manuscript. The manuscript was written through contributions of all authors.

## Conflicts of interest

All authors declare there are no conflicts of interest regarding this work.

## Supplementary Material

SU-004-D6SU00225K-s001

## Data Availability

All the data needed to support this study has been provided in the article and/or supplementary information (SI). Supplementary information: development of the pulping method used in this paper, peroxide bleaching tests on algal pulp, description of attempts on manufacturing paper from chemically untreated algal biomass, extended sugar analysis results, paper properties in numeric format, lab-scale proof of concept of HCl recycling of residual acid from algal pulping, the LCI of lab-scale algal papermaking, supplementary LCA figures to the main article and technical data on industrial HCl recycling in the context of the pickling industry with discussion of its application to algal papermaking. All of the above is compiled in a single report (PDF). Ref. 67–70 are cited only in the SI. Unprocessed raw data can be provided by the corresponding authors upon reasonable request. See DOI: https://doi.org/10.1039/d6su00225k.
