# Supplementary material for: Sustainable paper manufacture from Cladophora via hydrochloric acid process
Source: RSC Sustain. 2026 Sep 7;4(10):3703–13. doi: 10.1039/d6su00225k (PMC13563440; doi:10.1039/d6su00225k)
Supplement: Supplementary file 1 [file SU-004-D6SU00225K-s001.pdf]

Supplementary Information (SI) for RSC Sustainability.
This journal is © The Royal Society of Chemistry 2026

Supplementary Information: Sustainable Paper Manufacture from *Cladophora* via Hydrochloric Acid Process

Karl Mihhels [a], A. Topias Kilpinen [a], Neptun Yousefi [b*], Roozbeh Abidnejad [a], Jouni Paltakari [a], Erlantz Lizundia [c], Jaanika Blomster [d], and Eero Kontturi [a*]

[a] Department of Bioproducts and Biosystems, Aalto University, School of Chemical Engineering, 02150 Espoo, Finland
[b] Department of Chemical Engineering, Imperial College London, South Kensington Campus, Exhibition Rd., London SW7 2AZ, UK
[c] Life Cycle Thinking Group, Department of Graphic Design and Engineering Projects, Faculty of Engineering in Bilbao. University of the Basque Country (EHU), Bilbao 48013, Spain
[d] Jaanika Blomster, Department of Environmental Sciences, University of Helsinki, Finland

Table of contents

Supplementary information (SI) has been divided into 2 sections. First, the results and observations on papermaking made with *Cladophora glomerata (C. glomerata)* biomass and pulp are discussed, afterwards the technical details used to make the LCA are presented.

List of tables in the SI 2

List of figures in the SI 2

1. Papermaking trials with *Cladophora glomerata* 3-8

1.1 Identification of C. glomerata biomass during harvesting and processing 3

1.2 Preliminary hydrolysis and papermaking trials 3-4

1.3 Bleaching trials 4-6

1.4 Trials to produce paper directly from untreated *C. glomerata* algae 7

1.5 Determination of the basic properties of the acid filtrate 7

1.6 Sugar analysis of residual heteropolysaccharides 7-8

1.7 Numeric data for paper properties reported in the main article 8

1.8 Proof of concept of HCl-recycling via direct distillation 8-9

2. Life Cycle Assessment 10-16

2.1 LCI of the lab-scale algal papermaking 10-11

2.2 Supplementary data to the LCA presented in the main article 12-15

2.3 Considerations on HCl-recycling in the context of algal papermaking 15-16

List of tables in the SI

Table	Description	Page number
Table S1	Chemical loads of each sample point in the bleaching trial	5
Table S2	Monosaccharides present in the heteropolysaccharide fraction of algal papers.	7
Table S3	Numeric values for the paper property data presented in Fig. 3 of the main paper.	8
Table S4	Material and energy inventory of converting algal biomass to algal paper. (Life Cycle Inventory)	10-11
Table S5	Material inventory for lignocellulosic materials.	12
Table S6	*C. glomerata* raw biomass chemical composition, and detailed chemical composition of its ash fraction according to references	13
Table S7	Material inventory considering the potential credits upon integrated biorefinery implementation.	14
Table S8	Tabulation of the properties of the materials used in comparative LCA of Fig. 5 of the main work	14
Table S9	Summary table of the key LCA modelling assumptions	14-15

List of figures in the SI

Figure	Description	Page number
Figure S1	Hydrolysed alga after a 2 h reaction time.	3
Figure S2	Hydrolysed alga pulp after washing	4
Figure S3	Bleached alga pulp after filtration.	6
Figure S4	Paper sheet produced from bleached *Cladophora glomerata* alga pulp.	6
Figure S5	Untreated Cladophora biomass stuck to the papermaking equipment.	7
Figure S6	A setup for lab-scale distillation of HCl	9
Figure S7	Flowchart of converting algal biomass to algal paper.	10
Figure S8	Climate change potential of various paper products, based on Ecoinvent database versions (3.11 and 3.12).	12
Figure S9	*Cradle-to-grave* environmental impacts in eight relevant indicators according to the Environmental Footprint 3.1 impact assessment methodology.	13
Figure S10	Process flow diagram with field data of a HCl-recycling setup used in the metal industry. Republished with permission.[31]	15
Figure S11	Process flow diagram of a theoretical industrial process producing algal pulp and incorporating HCl-recycling	16

Number of Pages: 16

Number of Tables: 9

Number of Figures: 11

1. Papermaking trials with *Cladophora glomerata*

1.1 Identification of *C. glomerata* biomass during harvesting and processing

The following criteria were used to identify *C. glomerata*:

- It is a green filamentous alga that grows just below the waterline during the summer months (mainly June to September in the Baltic Sea).
- It forms dense monoclonal mats in the Baltic Sea that feel velvety to the touch when picked up. Structurally it usually forms tuft-like branched filaments.
- The only species that it can potentially be confused in the confines of the Tvärminne region of the Baltic Sea is Ulva intestinalis. Ulva intestinalis has a slippery tactile feel and a bright, translucent green appearance, making on-site differentiation while harvesting relatively easy. Structurally it usually forms tubular unbranching filaments.

Contamination from co-occurring algal species was minimised by harvesting from sites where *C. glomerata* was the visually dominant species, with residual contamination further reduced by the post-drying purification step described in the main text. The salinity of the waters in the harvesting area was not measured during harvesting, but is expected to be within the 0.45-0.7% range reported in the literature for the Tvärminne station.[67] Precisely quantifying salts within the biomass structure (e.g. salts having a biological function) versus surface-deposited salts originating from the dried seawater is methodologically difficult and was not attempted.

1.2 Preliminary hydrolysis and papermaking trials

Preliminary hydrolysis trials for *C. glomerata* were performed using 32% and 22% HCl (aq) with a 2 h reaction time. The acid was prepared according to the method described in the main article. These preliminary trials were conducted to identify a workable reaction window rather than to systematically optimise pulping conditions; the final concentration and reaction time were selected primarily to remain below the HCl azeotropic point (enabling acid recovery by distillation) and to permit safe handling at room temperature.

Algae samples were mixed in a rotary shaker at 200 rpm at room temperature (22°C) with acid-to-solids loading ratio of 12:1. The appearance of the hydrolysed algae after 2 h reaction is shown in Fig. S1.

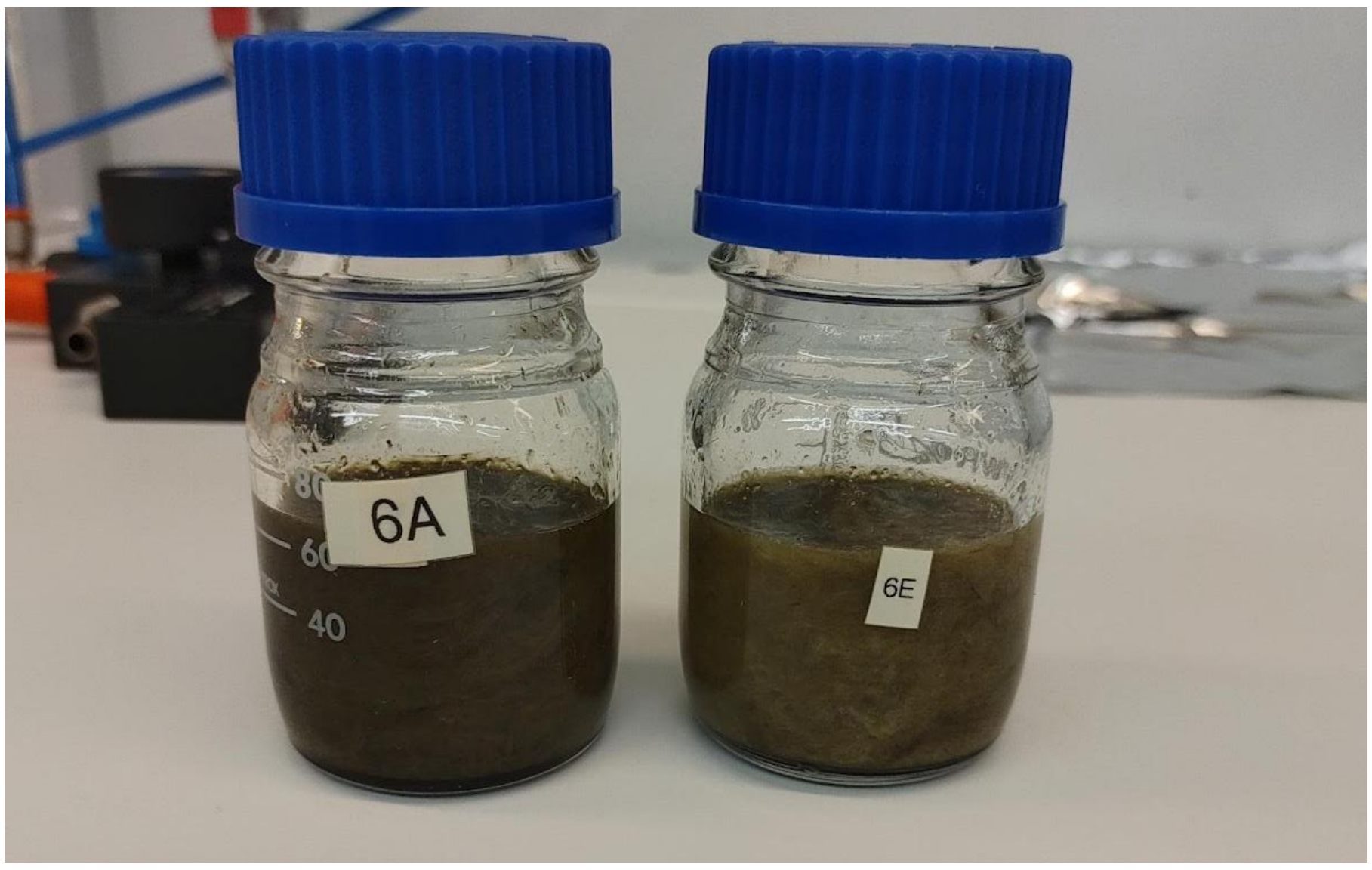

Fig. S1 *C. glomerata* biomass after 2 h of hydrolysis. Samples were treated with 32% HCl (aq) (left) and 22% HCl (aq) (right) at room temperature.

Following the 2 h reaction, the hydrolysis was quenched by adding 2 L of deionized water. The hydrolyzed algae were washed in a 200 µm mesh PE bag until pH 5. Washing was straightforward, with easy water flow through the fibre mat and no observation of gel-like plugs. While higher acid concentration more effectively removed

coloured compounds, a 20% concentration was selected for the main study to facilitate easier HCl recycling. The washed samples are illustrated in Fig. S2.

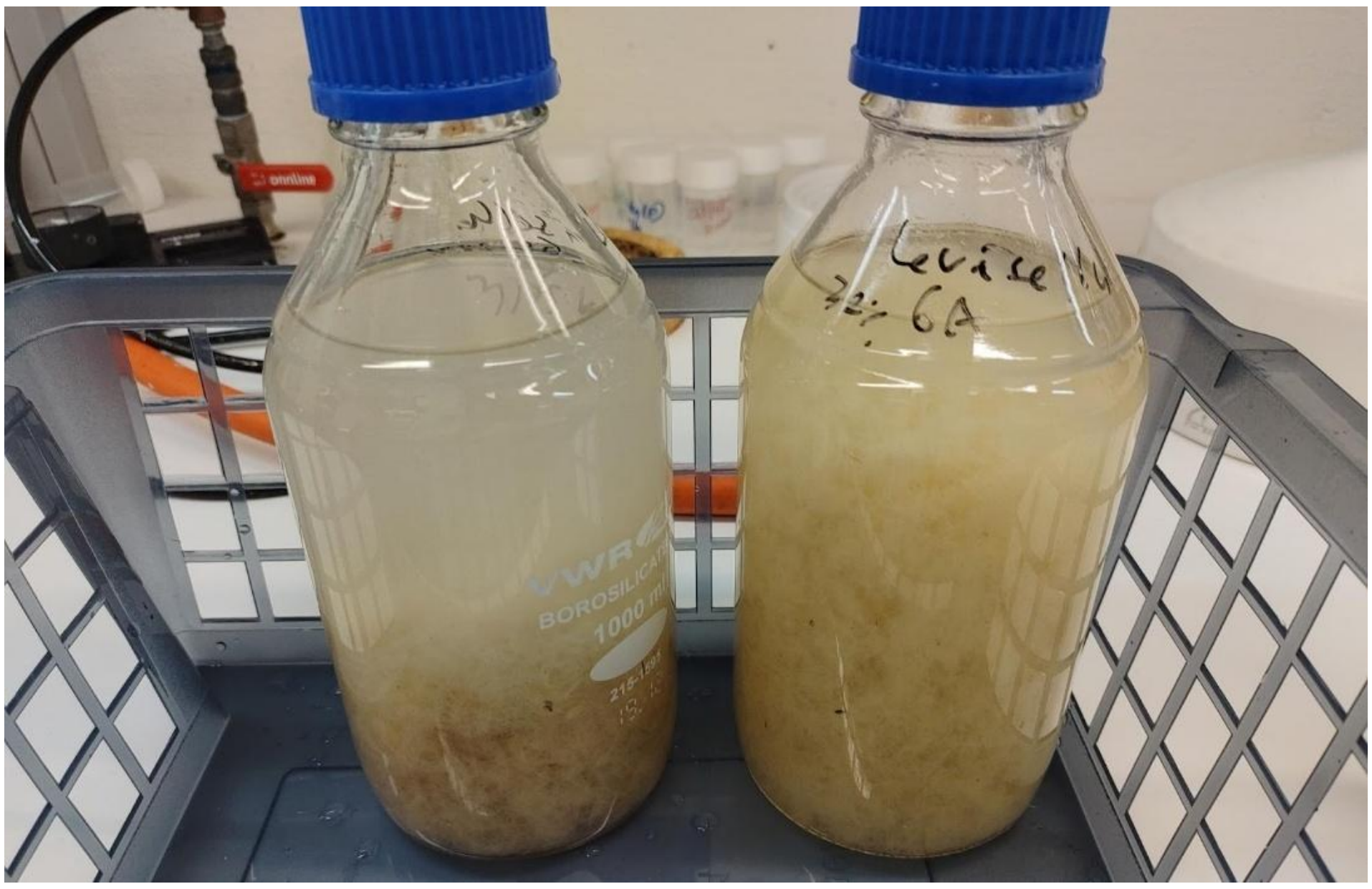

Fig. S2 Hydrolysed algae following the washing process. Samples were treated with 22% HCl (aq) (left) and 32% HCl (aq) (right).

From these initial test pulps papers were manufactured, but their properties were not recorded. At this stage pulp drainage was also qualitatively evaluated. Manufacturing thicker paper (~150 g/m^2) was possible, but extremely slow (drainage time in the order of minutes in the standard sheet former) due to the slow rate of water removal during paper sheet formation. However, if grammage remained relatively low (80 g/m^2 or less) HCl-treated *C. glomerata* fibres had adequate drainage and alterations to the standard papermaking processes were not needed. The Schopper-Riegler number measuring pulp drainage (°SR) was not determined as the singular value produced by this test was not considered to be meaningful without the establishment of baseline data, such as the beating and fibre-size microscopy studies found in the work of Chao et al. for rhizoclonium-based paper.[15]

Preliminary trials with alkaline cooking (1 M NaOH, 60 °C) were also carried out; these were not pursued further as a qualitative gel formation was observed that impeded washing, unlike with the acid-treated biomass.

1.3 Bleaching trials

The green colouration of unbleached algal paper (Fig. 1, main text) originates predominantly from chlorophyll and other pigments retained in the biomass; the trials below demonstrate that this colouration can be substantially reduced using conventional peroxide bleaching.

A set of bleaching trials was conducted to ascertain if *C. glomerata* pulp could be bleached using the same techniques used for wood. To prepare the samples, air-dry algae were hydrolysed with 32% HCl (aq) at 1:10 solid-to-liquid ratio for 2 h in a rotary shaker (200 rpm). Resulting pulp was diluted with deionized water (2 L), filtered (200 µm PE and then washed until pH 5. Excess water was squeezed out with hydraulic press (VARES, 2T) and dry matter content of the pressed pulp (DMC: 20%) measured.

For the bleaching of algal pulp, the experimental matrix presented in Table S1 was used. Bleaching was conducted by mixing the materials of each sample point in a plastic bag (LDPE) and immersing these bags in a water bath set to 70°C for 4 hours.

Table S1. Chemical loads of each sample point in the bleaching trial. Algae pulp used with 20% dry matter content.

**(A1)**<br>1.25 g algae pulp<br>0.28 g 35% H_2O_2<br>3.47 g deionized water	**(A2)**<br>1.25 g algae pulp<br>0.71 g 35% H_2O_2<br>3.04 g deionized water	**(A3)**<br>1.25 g algae pulp<br>1.42 g 35% H_2O_2<br>2.33 g deionized water	**(A4)**<br>1.25 g algae pulp<br>2.5 g 35% H_2O_2<br>1.25 g deionized water
**(B1)**<br>1.25 g algae pulp<br>0.28 g 35% H_2O_2<br>1.25 g 1 M NaOH<br>3.22 g deionized water	**(B2)**<br>1.25 g algae pulp<br>0.71 g 35% H_2O_2<br>1.25 g 1 M NaOH<br>2.79 g deionized water	**(B3)**<br>1.25 g algae pulp<br>1.42 g 35% H_2O_2<br>1.25 g 1 M NaOH<br>2.08 g deionized water	**(B4)**<br>1.25 g algae pulp<br>2.5 g 35% H_2O_2<br>1.25 g 1 M NaOH
**(C1)**<br>1.25 g algae pulp<br>0.28 g 35% H_2O_2<br>1.25 g 1 M NaOH<br>0.15 g sodium silicate<br>3.07 g deionized water	**(C2)**<br>1.25 g algae pulp<br>0.71 g 35% H_2O_2<br>1.25 g 1 M NaOH<br>0.15 g sodium silicate<br>2.64 g deionized water	**(C3)**<br>1.25 g algae pulp<br>1.42 g 35% H_2O_2<br>1.25 g 1 M NaOH<br>0.15 g sodium silicate<br>1.93 g deionized water	**(C4)**<br>1.25 g algae pulp<br>2.5 g 35% H_2O_2<br>1.25 g 1 M NaOH<br>0.15 g sodium silicate

After bleaching the samples were washed with deionized water. Circular sheets (seen in Fig. S3) were manufactured by deposition of the bleached pulp in a Büchner funnel on top of a cotton filter paper. After sheet formation, the sheets were detached from the cotton filter by hand and air-dried without support. Best whiteness (as seen in Fig. S3) was reached with treatments B4 and C4, but no direct measurements of brightness were made. This work was seen to be outside the scope of the main paper, but we consider it to be useful to share the observation that *Cladophora* biomass can be bleached in a manner comparable to traditional paper pulp bleaching methods.[68] The source of the colour is not in this case lignin, but the chromophores present in algal pulp seem to respond well to peroxide bleaching, especially in the presence of sodium silicate.

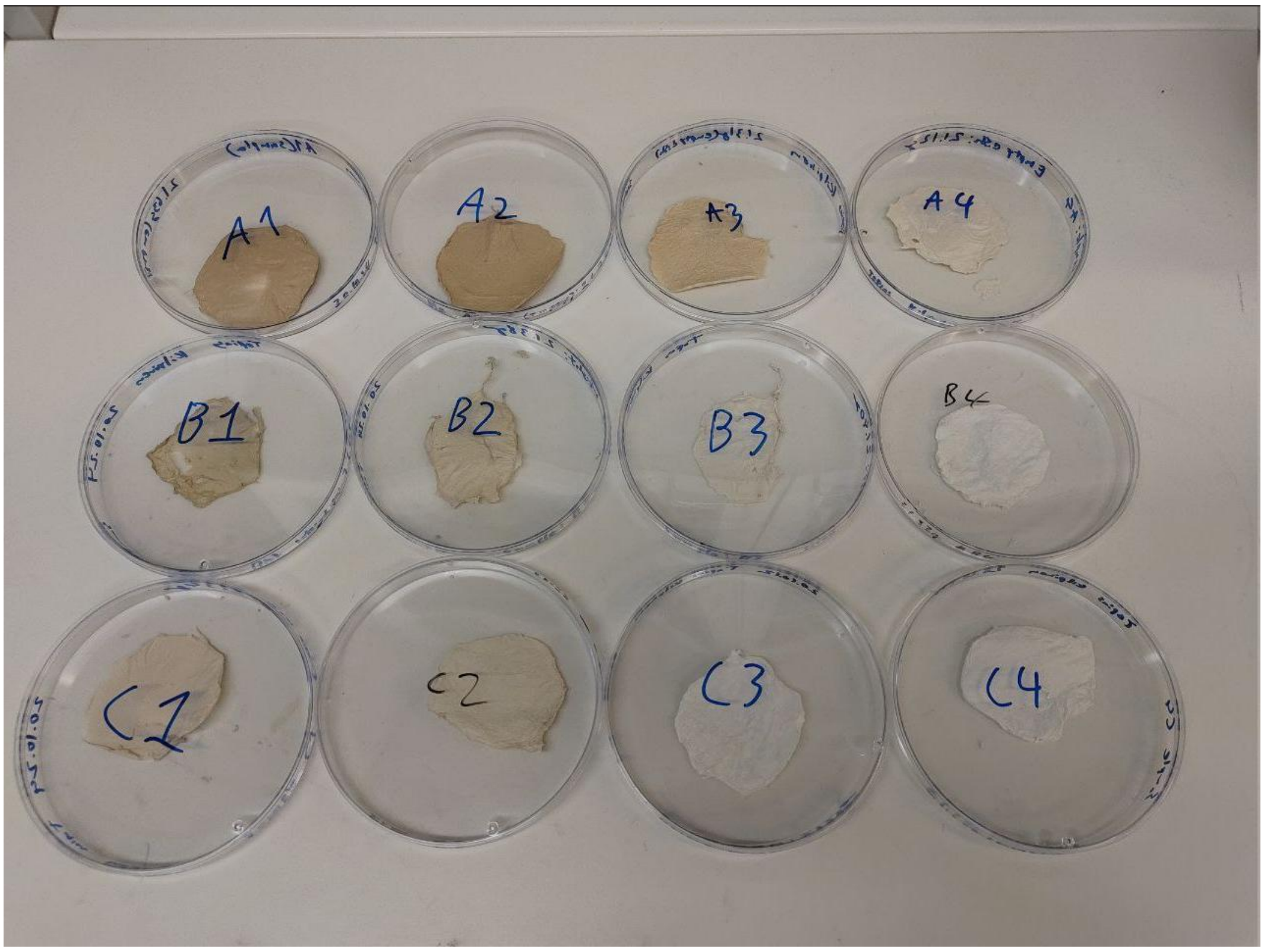

Figure S3 Circular sheets of bleached algae pulp after filtration and air-drying.

A further sample of pulp was bleached using the methodology of test point B4, and a low-grammage test paper sheet was made. This test paper could be written on without any problems with conventional marker- and ballpoint-pens, as demonstrated in Fig. S4. No direct paper quality measurements were made of this bleached paper sheet.

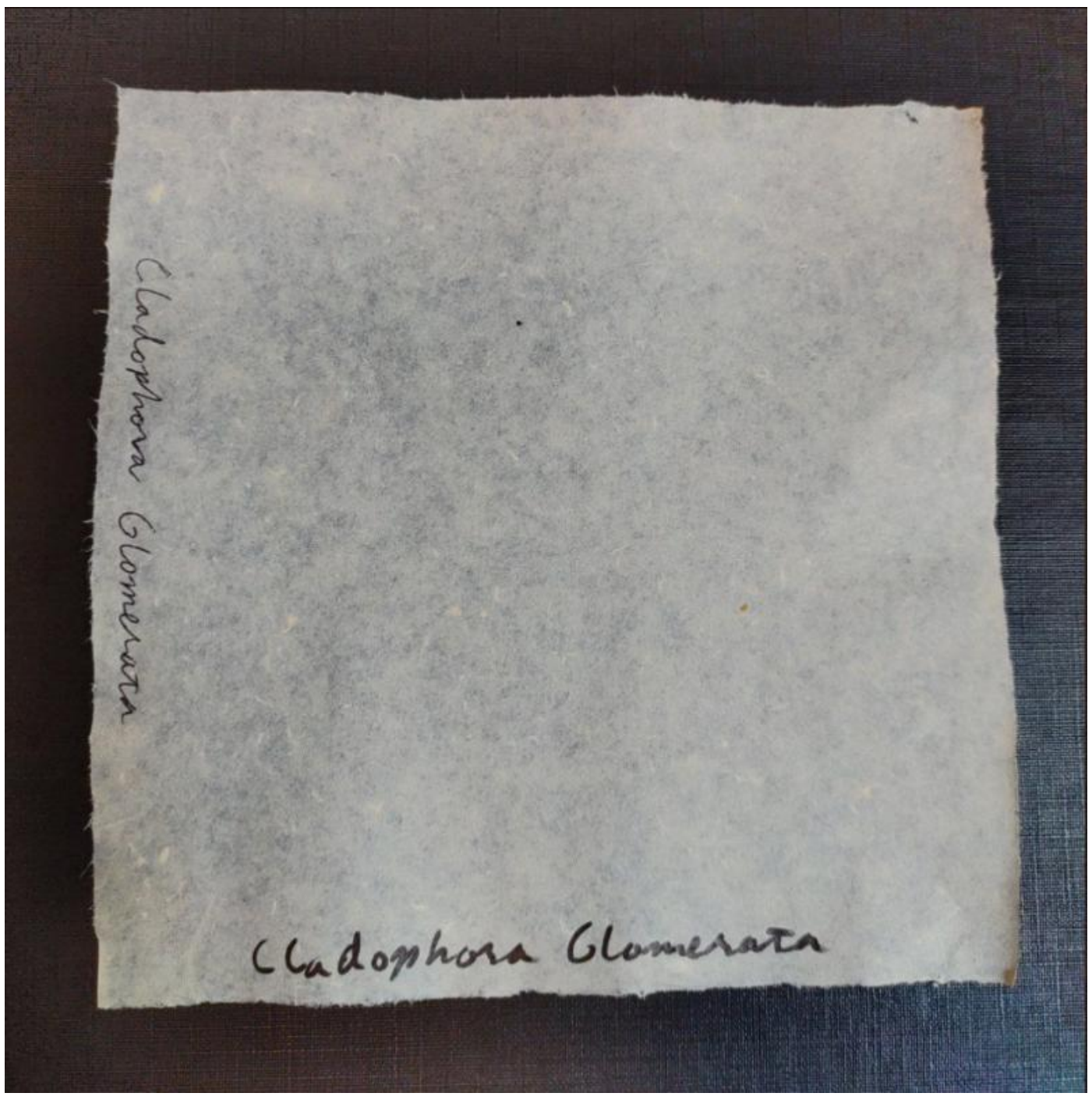

Figure S4 Paper sheet produced from bleached *C. glomerata* algal pulp.

1.4 Trials to produce paper directly from untreated *C. glomerata* algae

An attempt was made to produce paper directly from the *C. glomerata* algae, without any chemical processing, similarly to previous work.[11] Unfortunately, the untreated biomass proved to be extremely sticky, with fibres sticking to nearly all exposed process equipment surfaces used during papermaking. Removal of the formed sheets from the wire was challenging as the fibres attached to the forming wire, as seen in Fig. S5. In our experience, even though it was possible to manufacture some partial sheets, producing paper of consistent quality with good formation was not possible. This qualitative observation might explain why manufacturing of chemically untreated *C. glomerata* paper was not pursued further despite the good mechanical properties previously measured.[17]

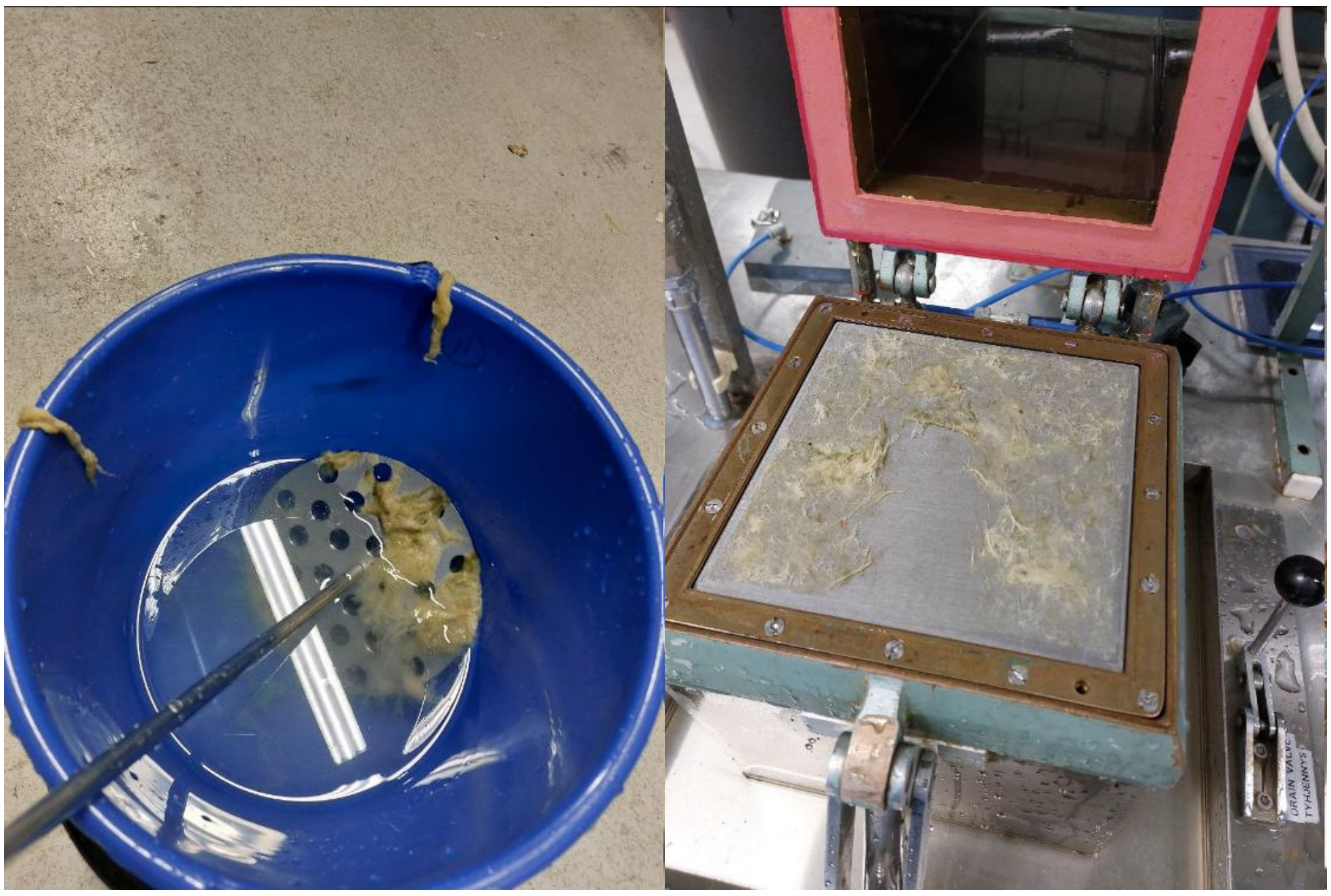

Figure S5 (left) Untreated *Cladophora* biomass stuck to the mixing equipment used in dispersing the fibres. (right) Tangled fibres of *Cladophora* biomass stuck to the forming wire after removal of a paper sheet.

1.5 Determination of the basic properties of the acid filtrate

The HCl used in pulping was titrated against 1 M NaOH (Titripur, Merck) in triplicate. The wt.% of the HCl used in this work to pulp the air-dry algal biomass was determined to be 19.6% ± 0.13 % (95% confidence). Similar titration was carried on the acid filtrate recovered directly after pulping, with the wt.% of HCl determined to be 18.5% ± 0.07 % (95% confidence).

The density of the acid filtrate was determined by filling a 10mL volumetric flask with well mixed filtrate and recording the weight. The measurement was done thrice, with a measured density of 1.109 g/mL ± 0.001 g/mL (95% confidence) for the acid filtrate.

1.6 Sugar analysis of residual heteropolysaccharides

The detailed monosaccharide profile of the algal papers manufactured in this study is presented in Table S2, which is referred to in the Table 1 of the main article. The heteropolysaccharide fraction presented in the main article is obtained by summation of all monosaccharides presented in Table S2. All measurements were conducted in duplicate and confidence intervals are reported at 95% value.

Table S2. Monosaccharides present in the heteropolysaccharide fraction of algal papers.

Paper sample	Xylose	Galactose	Arabinose	Mannose	Rhamnose
Alga 300	0.8 % ± 0.1 %	1.3 % ± < 0.1 %	0.5 % ± < 0.1 %	0.4 % ± < 0.1 %	0.8 % ± 0.1 %
Alga 10k	0.9 % ± < 0.1 %	1.3 % ± < 0.1 %	0.4 % ± < 0.1 %	0.3 % ± < 0.1 %	0.9 % ± 0.1 %

1.7 Numeric data for paper properties reported in the main article

Table S3. Numeric values for the paper property data presented in Fig. 3 of the main paper. 5 replicates used for samples "Alga 300" and "Alga 10k", and 10 for "MechPulp". Confidence interval is reported at 95% value.

Property	Alga 300	Alga 10k	MechPulp
Grammage (g/m^2)	108.6 ± 9.0	85.6 ± 2.8	81.1 ± 0.2
Thickness (µm)	159.0 ± 13.6	136.0 ± 9.9	185.0 ± 11.8
Density (kg/m^3)	682.6 ± 56.5	629.7 ± 20.7	439.0 ± 3.9
Bulk (m^3/kg)	1.5 ± 0.002	1.6 ± 0.05	2.28 ± 0.02
Bendtsen air permeability (mL/min)	29.9 ± 7.8	17.5 ± 1.9	76.5 ± 4.9
Opacity (%)	100 ± 0.02	99.8 ± 0.2	92.8 ± 0.2
ISO brightness	24.7 ± 0.2	25.3 ± 0.4	71.0 ± 0.4
Tensile strength (kN/m)	3.0 ±1.0	2.4 ±0.06	3.7 ± 0.1
Tensile stiffness index (kNm/g)	5.8 ± 0.6	4.4 ± 0.5	4.3 ± 0.2
Tensile Index (Nm/g)	27.3 ±2.4	28.6 ± 0.5	45.4 ± 1.7
Strain at break (%)	1.5 ±0.20	2.3 ± 0.3	3.2 ± 0.2
TEA (J/m2)	31.0 ±5.7	38.3 ± 6.3	82.2 ± 12.5
TEA Index (J/g)	0.28 ±0.08	0.46 ± 0.01	1.01 ± 0.15
Elastic modulus (GPa)	4.1 ± 0.5	2.2 ± 0.3	1.9 ± 0.2

1.8 Proof of concept of the HCl recycling via direct distillation

To justify the usage of HCl recycling a lab-scale experiment was conducted to test the applicability of the method used by Cunha et al.[30] to residual acid filtrate from algal pulping. The assembly of the equipment used for distillation is presented in Fig. S6. Of particular interest was to observe the behaviour of acid-dissolved algal solids during distillation, and if the distilled HCl would be contaminated with compounds emanating from this fraction of the original biomass.

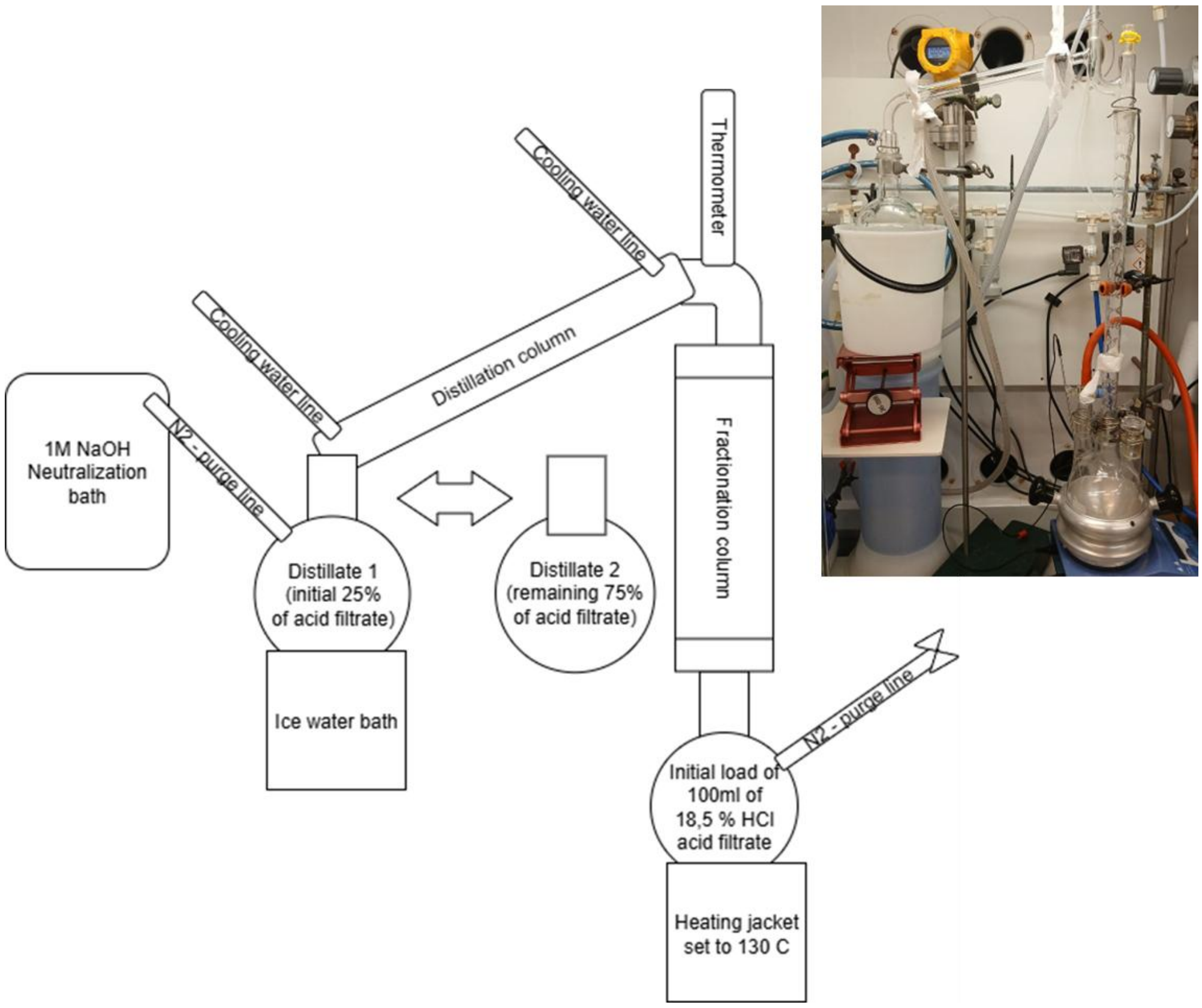

Figure S6 A setup for lab-scale distillation of HCl and acid-dissolved residues of algal biomass.

The distillation was separated into 2 stages. This was to find out if the distillation had qualitative differences while running well below the azeotrope point and near the azeotrope point. We halted the first distillation after 25% of the initial volume of the distillate in the system was recovered. After cooling down and purging the system with nitrogen, the second distillate was collected in another flask.

During the first distillation the initial 1-3 distillate drops contained volatile compounds originating from the biomass, identified by a brown tint present in the distillate. Subsequent distillate remained completely clear. During the second distillation the distillate remained completely clear throughout the distillation. After 15 min with no observable boiling present in the distillation bowl the distillation was terminated.

The residual solids in the distillation bulb most likely underwent humin formation or some degree of pyrolysis[69] as the reaction conditions are appropriate for this to happen and the biomass residue turned completely black in colour. At the end of the distillation a tar-like viscous liquid with char-like solid particles was recovered from the distillation bulb.

Distillation was effective in separating the acid-dissolved biomass solids from the HCl-water distillate. Due to this we believe that distillation similar to the methods used in the pickling industry[28-31] is a technically viable method for HCl-recovery in the context of this study. As a difference to the methods used in the pickling industry, the initial distillate containing unknown volatile compounds may be unsuitable for recycling and might need to be discarded or recovered in some other way. Also, there might have been some unobserved gas formation (e.g. CO or CO_2) due to degradation of biomass taking place during distillation, but this did not cause any visible problems for the operation of the distillation column.

A direct measurement quantifying the amount of recovered acid was not carried out as it would have required retooling of the distillation apparatus, as the N_2 purge line (shown in Fig. S6) connected to the neutralization bath did not have a valve. This lack of valve was justified for safety reasons (to avoid overpressure formation during distillation) but may have enabled partial neutralization of HCl gas at the neutralization bath interface and even evaporation of some water vapor from the neutralization bath to the distillation system.

2. Life Cycle Assessment

2.1 LCI of the lab-scale algal papermaking

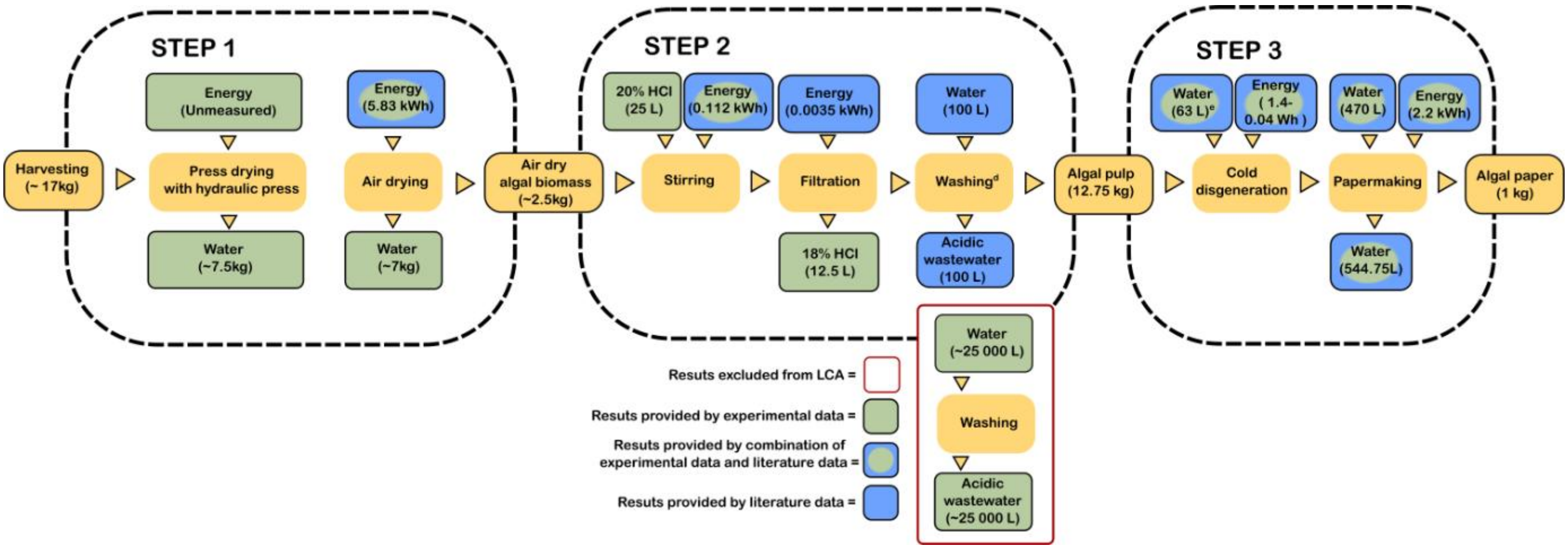

Figure S7 Flowchart of converting algal biomass to algal paper used as a basis for making the LCI.

Table S4. Material and energy inventory of converting algal biomass to algal paper. Wastes are highlighted in green. Process output is highlighted in blue.

Comment	Item	Amount	Source/provider
Step 1: Harvesting and preservation			
Cellulose source	Harvesting *Cladophora glomerata* (wet weight / bone-dry weight) [a]	17.0 kg / 2.255 kg	2.75% of the impacts of *Ulva lactuca*, from Agribalyse 3.1.1 database
	Transport (100 km, Tvärminne aquatic research center to Helsinki)	2.55 t × km	market for transport, freight, lorry, 3.5-7.5 metric ton, diesel, EURO 6 | transport, freight, lorry, 3.5-7.5 metric ton, diesel, EURO 6 | Cutoff, U - RER
Press-drying	Energy	5.80 kWh	market for electricity, medium voltage, renewable energy products | electricity, medium voltage, renewable energy products | Cutoff, U - CH
Water removed by press filtration	Wastewater	7.50 L	treatment of wastewater, average, wastewater treatment | wastewater, average | Cutoff, U - Europe without Switzerland
Water removed by evaporative/press-drying	Water vapor	7.50 L	Release to air
-	Air-dry biomass (wet weight / bone-dry weight)	2.50 kg / 2.255 kg	*Process Output*
Step 2: acid hydrolysis			
For acid hydrolysis, 1 L at 19.6 wt.% HCl per 100 g air-dry algae	Water	8.33 L	tap water production, conventional treatment | tap water | Cutoff, U - Europe without Switzerland
	HCl (as neat 5.73 kg)	19.10 L	market for hydrochloric acid, without water, in 30% solution state | hydrochloric acid, without water, in 30% solution state | Cutoff, U - RER

Electricity for stirring	Energy [b]	0.112 kWh	market for electricity, medium voltage, renewable energy products | electricity, medium voltage, renewable energy products | Cutoff, U - CH
Electricity for filtration	Energy [c]	0.0035 kWh	market for electricity, medium voltage, renewable energy products | electricity, medium voltage, renewable energy products | Cutoff, U - CH
Pulp washed with tap water until neutrality in a wire mesh bag	Water [d]	100 L	tap water production, conventional treatment | tap water | Cutoff, U - Europe without Switzerland
Acid with dissolved solids from the reaction	Acid wastewater	100 L	treatment of wastewater, average, wastewater treatment | wastewater, average | Cutoff, U - Europe without Switzerland
To neutralize 18.5 m HCl acid filtrate	Neutralizing agent	12.26	soda ash, light, crystalline, heptahydrate, to generic market for neutralising agent | neutralising agent, sodium hydroxide-equivalent | Cutoff, U - RER
-	Wet algal pulp (wet weight / bone-dry weight)	12.75 kg / 1.00 kg	*Process Output*
Step 3: processing into paper			
Tap water to dilute at 1.5 wt.% for disgeneration	Water	63 L	tap water production, conventional treatment | tap water | Cutoff, U - Europe without Switzerland
Electricity for cold-disgeneration	Energy [e]	1.375 Wh	market for electricity, medium voltage, renewable energy products | electricity, medium voltage, renewable energy products | Cutoff, U - CH
Papermaking water consumption	Water [g]	470 L	tap water production, conventional treatment | tap water | Cutoff, U - Europe without Switzerland
Electricity for papermaking and drying	Energy [f]	2.2 kWh	market for electricity, medium voltage, renewable energy products | electricity, medium voltage, renewable energy products | Cutoff, U - CH
-	Wastewater	544.75 L	treatment of wastewater, average, wastewater treatment | wastewater, average | Cutoff, U - Europe without Switzerland
Dry mass	Algal-paper	1.00 kg	*Process output*

[a]: The biomass acquisition is modelled as 2.75% of the impacts of *Ulva lactuca* (sea lettuce) for food consumption, which is a green algae similar to *Cladophora glomerata*.
[b]: Orbital shaker similar to OHAUSE SHHD1619AL used for laboratory work. For scaling calculations OHAUSE SHHD4550DG with a Motor power input of 80 W is used with a 70% workload. For manufacturer technical sheets, see: https://dmx.ohaus.com/WorkArea/showcontent.aspx?id=4294987925, Accessed 3.11.2025.
[c]: Büchner filtration facilitated by a diaphragm pump. Motor power 60 W used with a 70% workload. Typical filtration time is 5 minutes. For manufacturer technical sheets, see: https://assets.fishersci.com/TFS-Assets/CCG/EU/Welch-Vacuum-Technology/brochures/12139%20FB%20Vacuum%20pumps_EN.pdf , Accessed 3.11.2025.
[d]: 8 L of water / 1 L of reaction medium is assumed for efficient washing considering a non-optimised scenario (Ratio of 2.5 of water to reaction medium should be sufficient to wash most wood pulps.
[e]: Water consumption estimated from experimental work following standard ISO 5263-2.[36]
[f]: Instrument power use is 250 Wh. Power consumption has been multiplied by 25 to account for the size of functional unit compared to the batch size of the instrument.
[g]: Water consumption estimated from experimental work following standard ISO 5269-1.[37]
[h]: A representative energy consumption at a paper mill (2.2 MWh/t) has been considered.

2.2 Supplementary data to the LCA presented in the main article

Results quoted or calculated using references are reported as midpoint indicators throughout, rather than normalised against generic global or continental reference values, as normalisation can introduce additional assumptions and artefacts that reduce the transparency and comparability of individual impact categories.

Table S5 and Fig. S8 refer to the sources used to estimate the climate change potential of wood-based papers in Fig. 5B and 5C of the main paper. To complement the LCA analysis using CO_2-eq.$\cdot$kg^{-1} Fig. S9 compares lab-scale algal paper production to production of multiple different paper grades in eight environmentally relevant indicators. Data from tables S6 and S7 has been used to create the "Credits co-products" – scenario in Fig. 6 of the main paper.

Table S5. Material inventory for lignocellulosic materials.

Item	Source/provider
Tissue paper	tissue paper production, virgin | tissue paper | Cutoff, U - RER
Newsprint paper	paper production, newsprint, virgin | paper, newsprint | Cutoff, U - RER
Woodfree paper	paper production, woodfree, uncoated, at non-integrated mill | paper, woodfree, uncoated | Cutoff, U - RER
Supercalendered paper	paper production, wood containing, supercalendered | paper, wood containing, supercalendered | Cutoff, U - RER
Graphic paper	graphic paper production, 100% recycled | graphic paper, 100% recycled | Cutoff, U - RER
Kraft paper	kraft paper production | kraft paper | Cutoff, U - RER

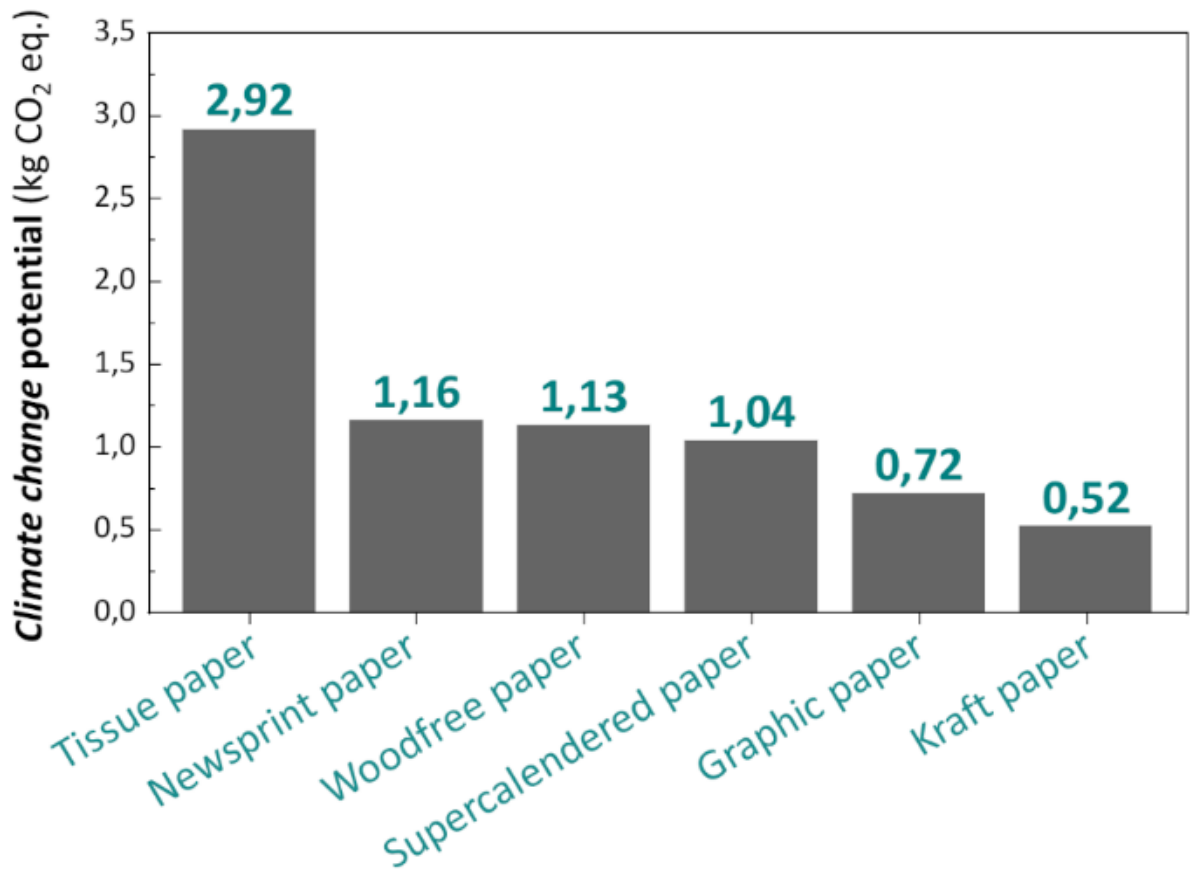

Figure S8 Climate change potential of various paper products, with based on Ecoinvent database (3.11 and 3.12).

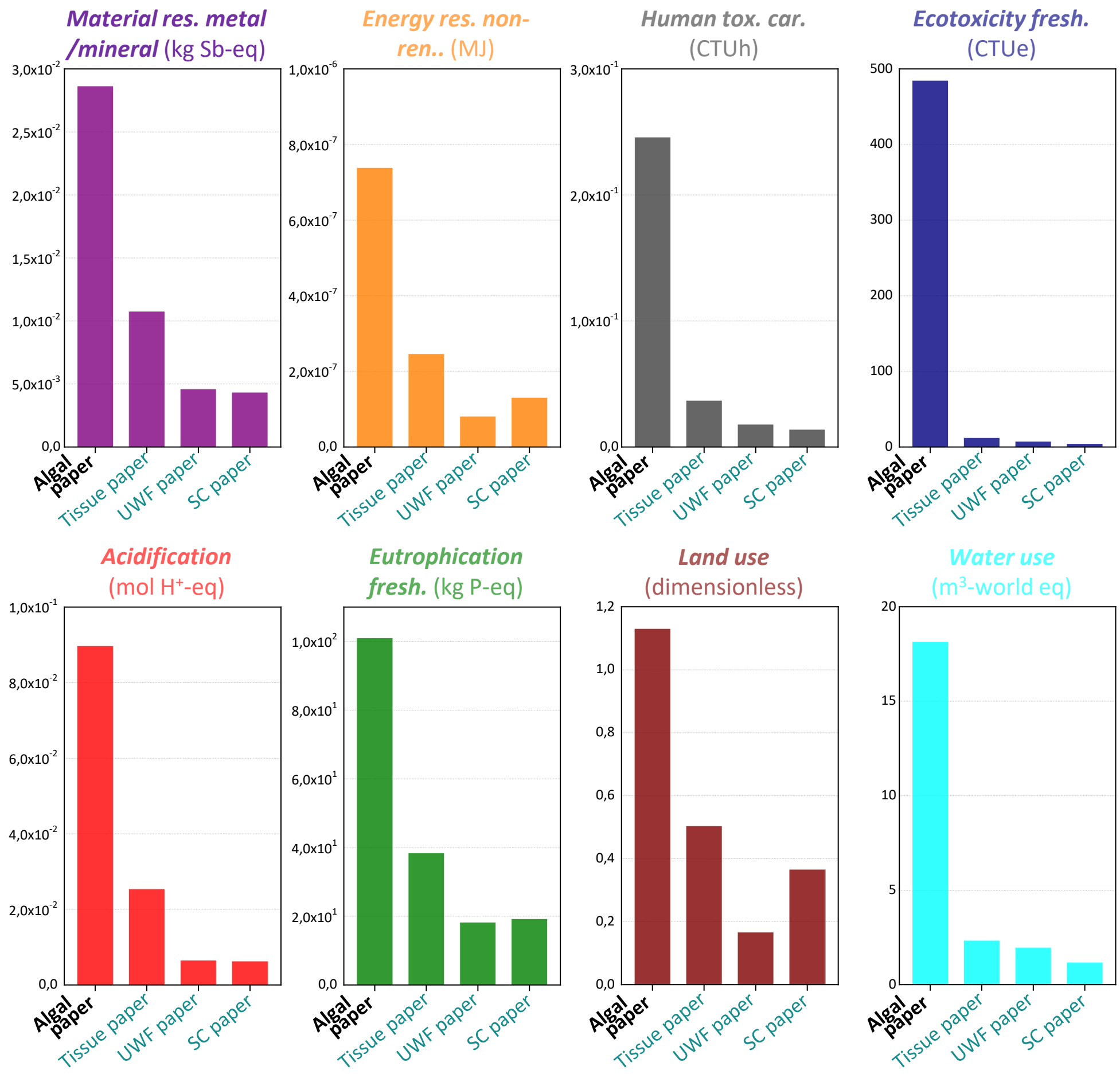

Figure S9 *Cradle-to-grave* environmental impacts in eight relevant indicators according to the Environmental Footprint 3.1 impact assessment methodology. The results are compared with the impacts obtained for three competitive lignocellulosic paper materials.

Table S6. *Cladophora glomerata* raw biomass chemical composition, and detailed chemical composition of its ash fraction according to references.

**Raw *Cladophora glomerata* biomass composition**[11,70]	**As % of dry weight**
Hemicelluloses	14-18 %
Cellulose	38-42 %
Proteins	6-12 %
Ash	16-24 %
Undetermined	6-15 %
**Detailed composition of ash from raw *Cladophora glomerata***[70]	**As % of ash**
$CaCO_3$	67 %
Undetermined ash	18 %
SiO_2	11 %
MgO	3 %
Al_2O_3	1 %
Na	< 1 %
K	< 1 %

Table S7. Material inventory considering the potential credits upon integrated biorefinery implementation. Data correspond to a single batch at laboratory scale, yielding a paper of 40 grams.

Comment	Item	Amount (g)	Source/provider
Not recovered due to low economic value	Hemicelluloses	-	-
-	Proteins	10.82	Protein pea production | protein pea | Cutoff, U - RoW
Ammonium sulfate is selected because of the large N content of Cladophora glomerata	Sulfates	6.31	Ammonium sulfate production | ammonium sulfate | Cutoff, U - RER
Ashes	$CaCO_3$	9.63	Calcium carbonate production, precipitated | calcium carbonate, precipitated | Cutoff, U - RER
	SiO_2	1.63	Silica sand production | silica sand | Cutoff, U - RoW

Table S8. Tabulation of the properties of the materials used in comparative LCA of Fig. 5 of the main work. The environmental impacts have been extracted from the *"ecoinvent v3.11 Cut-Off Unit Processes"* database, utilizing the Environmental Footprint v3.1 impact assessment method. Extrusion was selected as the processing approach for such materials, and the utilized elastic modulus and density for calculations are listed in the table.

Material	Density (kg/L)	Elastic modulus (GPa)
ABS	1.050	2.588
PP	0.904	1.325
PET	1.375	2.950
LDPE	0.919	0.300
PC	1.210	2.200
PLA (Natureworks)	1.240	3.600
PVC	1.450	3.275
PMMA (Natureworks)	1.185	2.855

Table S9. Summary table of the key LCA modelling assumptions

**Category**	**Assumption**	**Basis / Location**
**System boundary**	Cradle-to-grave: raw material sourcing, on-site electricity, waste management, end-of-life	Section 2.3
**Equipment amortisation**	Excluded from the model	Section 2.3
**Functional units**	(1) 1 kg of material;<br>(2) impact normalised per specific tensile strength (density*EIp / tensile modulus)	Section 2.3, Eq. 1
**Electricity source**	EU renewable medium-voltage electricity mix, aligned with EU/2023/2413 renewable-energy targets	Section 2.3
**Background databases**	ecoinvent v3.11 Cut-Off Unit Processes (Dec 2024); Agribalyse 3.1.1 (Jan 2024)	Section 2.3
**Impact assessment method**	Environmental Footprint v3.1, calculated in OpenLCA 2.5	Section 2.3
**Co-product handling**	System expansion with substitution, per ISO 14044	Section 2.3

**Raw algal biomass burden**	Not zero-burden; modelled as 2.75% of impacts reported for food-grade Ulva lactuca cultivation, without biogenic carbon accounting	Section 3.2.1
**Washing water ratio**	3.6 × reaction volume, chosen as a conservative estimate above the ~2.5x industrial displacement-washing benchmark	Section 2.1.1, 2.3
**Acid recycling / HCl recovery**	Not included in the base-case LCA; evaluated separately via a sensitivity scenario using pickling-industry field data	Section 3.2.2, Fig. 6
**Benchmark wood-based papers**	Sourced from ecoinvent (tissue, SC, UWF paper); benchmark plastics from ecoinvent + Agribalyse, extrusion assumed as processing route, with specified density/elastic modulus per material	SI Table S5, S7
**Allocation sensitivity**	Evaluated across five scenarios: original, no-neutralisation, algae burden-free, algae food-grade, co-product credit	Fig. 6

2.3 Considerations on HCl-recycling in the context of algal papermaking

Implementation of a pickling-type HCl recycling system in the context of algal papermaking should take into account the considerations relevant for industrial scale production processes. While our lab-scale distillation test can be considered a proof-of-concept, it does not reflect the realities of industrial HCl recycling very well.

Fig. S10 is an extended process flow diagram of an industrial suppliers HCl-evaporative recovery system. In this system, the distillation is not carried out until completion. Instead the system has been optimised for minimizing energy consumption in respect to the amount of concentrated HCl recovered. The recovery of residual HCl leaving with the "Metal Conc" and "Distillate" – flow(s) in Fig. S10, is considered by the supplier to be economically unjustified. The material and energy flows presented in Fig. S10 are in our opinion a realistic estimate of a state-of-the-art HCl recycling system and thus serve as a good baseline estimate for energy intensiveness of HCl recycling.

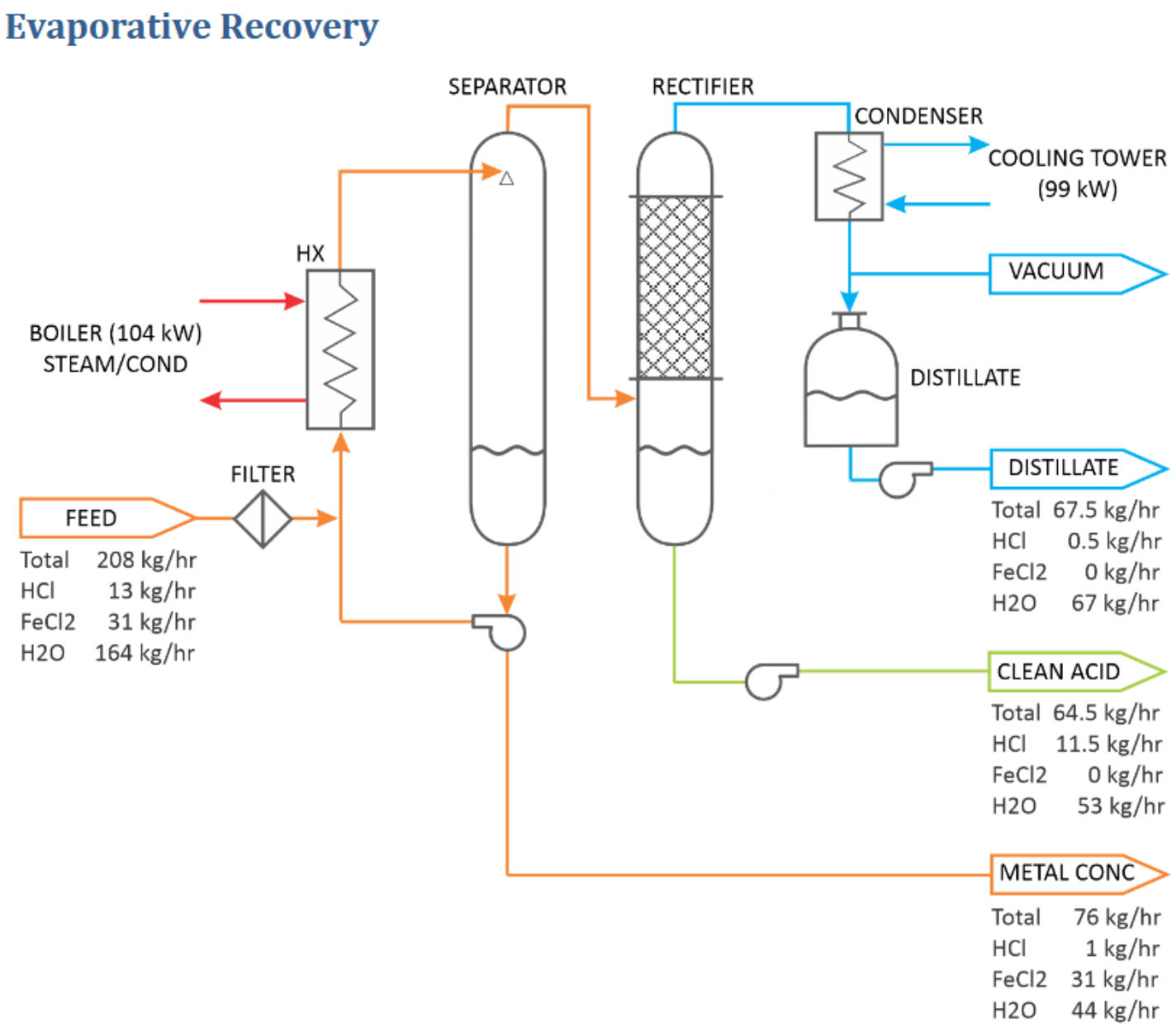

Figure S10 Extended process flow diagram of an industrial suppliers HCl-evaporative recovery system. Numerical values provided by field data of the supplier. Image republished with permission.[31]

In Fig. S11 a concept-level process diagram is presented to demonstrate how a fictional algal paper mill would use the existing HCl recycling technology as unit operation. The process area covered by the minimum estimate of 1 CO2-eq.·kg^{-1} presented in the main paper is circled with red. The separation unit for dissolved solids and neutralization tank have been left outside the considerations of LCA (and are discarded from being system extensions) as the HCl recycling is estimated to be the most energy and resource intensive operation and the choice of best available technologies in these other operations is less clear. Despite these inaccuracies, we estimate that the total CO_2-impact of the process, even if the previously mentioned system extensions would be included, would be well below the 5.61 CO_2-eq.·kg^{-1}, the impact of direct neutralization.

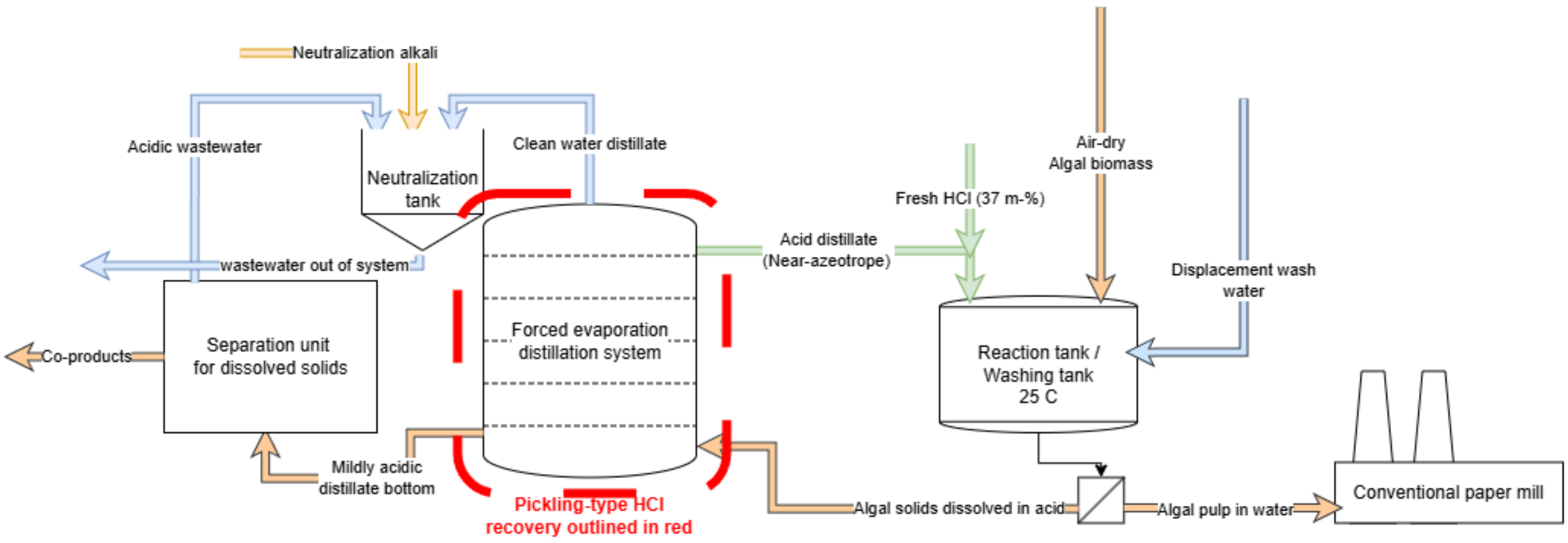

Figure S11 Process flow diagram of a theoretical industrial process producing algal pulp and incorporating pickling-type HCl-recycling.
